# Asian Sand Dust Particles Increased Pneumococcal Biofilm Formation *in vitro* and Colonization in Human Middle Ear Epithelial Cells and Rat Middle Ear Mucosa

**DOI:** 10.3389/fgene.2020.00323

**Published:** 2020-04-24

**Authors:** Mukesh Kumar Yadav, Yoon Young Go, Sung-Won Chae, Moo Kyun Park, Jae-Jun Song

**Affiliations:** ^1^Institute for Medical Device Clinical Trials, Korea University College of Medicine, Seoul, South Korea; ^2^Department of Biotechnology, Pachhunga University College, Mizoram Central University, Aizawl, India; ^3^Department of Otorhinolaryngology-Head and Neck Surgery, Korea University College of Medicine, Seoul, South Korea; ^4^Department of Otorhinolaryngology-Head and Neck Surgery, Seoul National University College of Medicine, Seoul, South Korea

**Keywords:** Asian sand dust, *Streptococcus pneumoniae*, biofilms, colonization, otitis media, RNA-sequencing

## Abstract

**Introduction:**

Air pollutants such as Asian sand dust (ASD) and *Streptococcus pneumoniae* are risk factors for otitis media (OM). In this study, we evaluate the role of ASD in pneumococcal *in vitro* biofilm growth and colonization on human middle ear epithelium cells (HMEECs) and rat middle ear using the rat OM model.

**Methods:**

*S. pneumoniae* D39 *in vitro* biofilm growth in the presence of ASD (50–300 μg/ml) was evaluated in metal ion-free BHI medium using CV-microplate assay, colony-forming unit (cfu) counts, resazurin staining, scanning electron microscopy (SEM), and confocal microscopy (CF). Biofilm gene expression analysis was performed using real-time RT-PCR. The effects of ASD or *S. pneumoniae* individually or on co-treatment on HMEECs were evaluated by detecting HMEEC viability, apoptosis, and reactive oxygen species (ROS) production. *In vivo* colonization of *S. pneumoniae* in the presence of ASD was evaluated using the rat OM model, and RNA-Seq was used to evaluate the alterations in gene expression in rat middle ear mucosa.

**Results:**

*S. pneumoniae* biofilm growth was significantly (*P* < 0.05) elevated in the presence of ASD. SEM and CF analysis revealed thick and organized pneumococcal biofilms in the presence of ASD (300 μg/ml). However, in the absence of ASD, bacteria were unable to form organized biofilms, the cell size was smaller than normal, and long chain-like structures were formed. Biofilms grown in the presence of ASD showed elevated expression levels of genes involved in biofilm formation (*luxS)*, competence (*comA*, *comB*, *ciaR)*, and toxin production (*lytA* and *ply)*. Prior exposure of HMEECs to ASD, followed by treatment for pneumococci, significantly (*P* < 0.05) decreased cell viability and increased apoptosis, and ROS production. *In vivo* experiment results showed significantly (*P* < 0.05) more than 65% increased bacteria colonization in rat middle ear mucosa in the presence of ASD. The apoptosis, cell death, DNA repair, inflammation and immune response were differentially regulated in three treatments; however, number of genes expressed in co-treatments was higher than single treatment. In co-treatment, antimicrobial protein/peptide-related genes (S100A family, Np4, DEFB family, and RATNP-3B) and OM-related genes (CYLD, SMAD, FBXO11, and CD14) were down regulated, and inflammatory cytokines and interleukins, such as IL1β, and TNF-related gene expression were elevated.

**Conclusion:**

ASD presence increased the generation of pneumococcal biofilms and colonization.

## Introduction

Otitis media (OM) is inflammation of the middle ear that affects children and elders (Monasta et al., 2012; Qureishi et al., 2014; Byeon, 2019). The recurrent OM adversely affects speech, hearing ability, and language development in children, finally leading to hearing loss (Bellussi et al., 2005; Roditi et al., 2016). Worldwide, OM-related complications result in approximately 21,000 deaths annually, and 65–300 million individuals are affected by chronic OM (COM) (Acuin, 2004). The incidence and prevalence of OM are particularly high in the Asian population (8.12–14.52%), which affect the quality of life and pose a significant social and economic burden (Monasta et al., 2012; Coleman et al., 2018; Byeon, 2019). OM is generally considered a multi-factorial abnormality of the middle ear, with many associated risk factors, including microbial exposure, environment, immunological deficiency, gender, and age (Jensen et al., 2013; Coleman et al., 2018). Among microbial agents, *Streptococcus pneumoniae* (*S. pneumoniae*) is an important commensal bacterium that colonizes asymptotically and causes infection under immune-compromised conditions (Coleman et al., 2018). Despite the introduction of a 13-valent vaccine in various countries to control pneumococcal infection, pneumococci remain the leading pathogen (Milucky et al., 2019). Moreover, *S. pneumoniae* forms biofilms, and its direct detection from biopsy samples of the middle ear mucosa of children with COM (Hall-Stoodley et al., 2006) suggests that pneumococcal biofilms are important virulence factors of OM (Blanchette et al., 2016; Vermee et al., 2019). In the nasopharyngeal cavity, pneumococci initially colonize and form biofilms that serve as a reservoir, and then, the biofilm bacteria can transit to other sterile sites causing OM, meningitis, pneumonia, bacteremia, and sepsis in immune-compromised individuals (children and elderly) (Chao et al., 2015; Weiser et al., 2018).

Recent studies have reported that particulate matter (PM) involved in air pollution is an important risk factor for OM (Park et al., 2018). Air pollution due to sand and dust storms originating in arid and semi-arid regions affects 151 countries worldwide (Middleton and Kang, 2017). A seasonal episode of Asian sand dust (ASD), which originates from arid areas of Mongolia and the Gobi desert, and affects the Korean peninsula, Japan, and China, is a major source of pollution in the East and Northeast Asia (Jung, 2016). In recent years, a growing number of reports have suggested that ASD exposure negatively affects human health, leading to respiratory diseases (Jung et al., 2012; Yu et al., 2012; Watanabe et al., 2016; Nakao et al., 2018) and significant mortality annually (Chen et al., 2004; Lee et al., 2014; Al et al., 2018). In addition, ASD contains PM of diameters < 10 μm (PM_10_) along with inhalable hazardous chemical components such as sulfate (SO_4_^2–^) and nitrate (NO_3_^–^) and microbes such as bacteria, viruses, and fungi (Chen et al., 2010). One study has reported a positive association between early exposure to PM pollution and OM (Kennedy et al., 2018). Recently, a study reported the implication of air pollution containing PM_10_, nitrogen dioxide (NO_2_), ozone (O_3_), sulfur dioxide, and carbon monoxide in OM (Park et al., 2018). Similarly, Girguis et al. (2018) suggested that preterm infants are most susceptible to infant bronchiolitis and OM associated with acute PM_2_._5_ exposure (Girguis et al., 2018). Several studies have reported that air pollution and nasopharyngeal bacteria are two known risk factors for OM (Chao et al., 2015; Park et al., 2018; Weiser et al., 2018). However, the interaction between these two factors remains unknown. Most research to date has focused on the implication of either *S. pneumoniae* or air pollutants in causing OM. Indeed, severe pneumonia and OM were reported in cigarette smokers exposed to PM and bacteria such as *S. pneumoniae* and *Haemophilus influenzae* (Nuorti et al., 2000; Givon-Lavi et al., 2006). In the nasopharynx, pneumococci and PM interact, and PM exposure increases the risk of infections and alters the function of native immune cells, impairs mucociliary clearance, and decreases phagocytosis by macrophages (Becker and Soukup, 1999; Zelikoff et al., 2002; Watanabe et al., 2016). PM including environmental tobacco smoke increases the risk of OM in children and changed the middle ear histology and eustachian tube mucosa (Kong et al., 2009; Jones et al., 2012; Bowatte et al., 2018; Nakao et al., 2018; Park et al., 2018). Previously, our group identified a potential biomarker and a signaling pathway related to OM induced by diesel exhaust particles (Kim et al., 2016). We also demonstrated that ASD exposure decreases the cell viability in a model human middle ear epithelium cell (HMEEC); affects apoptosis, cell proliferation, and oxidative stress-related gene expressions; and induces inflammatory responses in the rat middle ear epithelium (Go et al., 2015; Chang et al., 2016). However, the interaction, colonization, and virulence of *S. pneumoniae* in the presence of ASD are not known. Previous reports suggested that PM alters host immune defense (Becker and Soukup, 1999; Zelikoff et al., 2002; Watanabe et al., 2016) and induces OM (Bowatte et al., 2018; Nakao et al., 2018; Park et al., 2018); therefore, we hypothesized that the presence of a small quantity of ASD may increase pneumococcal *in vitro* biofilms, colonization to epithelial cells and middle ear mucosa, and the risk factor for pneumococcus-mediated OM. In this study, we evaluated the effect of ASD on *S. pneumoniae in vitro* biofilm growth, colonization on HMEECs, and rat middle ear using the rat OM model.

## Materials and Methods

### Ethics Statement

The animal study was approved by the Institute Review Board (IRB) of Korea University, Seoul, South Korea, with IRB approval number KOREA-2019-0029. The animal protocols were approved by Institutional Animal Care and Use Committees (IACUCs), Korea University, Seoul.

### Bacteria Strain and Culture Medium

In this study, *S. pneumoniae* D39, Avery’s virulence serotype 2 strain (NCTC; Salisbury, United Kingdom) was used (Avery et al., 1944). This strain is fully sequenced and well characterized and has remained highly virulent in the animal model after many years of its isolation. *Pseudomonas aeruginosa* (PA01) and *E. coli* (ATCC 24213) were purchased from ATCC, United States, and MRSA (CCARM 3903) was purchased from the culture collection of antimicrobial resistant strain (CCARM), Seoul, South Korea. Bacteria were grown in BHI broth, and glycerol stocks were maintained at −80°C. Pneumococci colonies were grown on a blood agar plate (BAP) supplemented with 5% sheep blood (Yang Chemical, Seoul, South Korea). The ASD particles used in this study were collected and composition was detected in our previous study (Chang et al., 2016). Briefly, ASD was collected using a high volume air sampler (HV500F, Sibata, Tokyo, Japan) at a flow rate of 500 L/minute, pre-filtered into filter packs (Prefilter AP, 124 mm; EMD Millipore, Bedford, MA, United States), and sieved through a filter with a 10-μm pore size (Chang et al., 2016). The stock solution of ASD was prepared in PBS and sonicated and sterilized using an autoclave.

### Planktonic Growth and *in vitro* Biofilm Growth

To study the effects of ASD on bacterial growth, *S. pneumoniae* planktonic growth was evaluated with different concentrations of ASD (50, 150, and 300 μg/ml). The pneumococcal growth was detected by measuring optical density at 600 nm (OD_600_) at different time points. *S. pneumoniae* planktonic growth was also evaluated by detecting metabolically active bacteria by resazurin staining after 48 h. Bacteria were grown in metal ion-free BHI medium based on a previous report (BHI medium treated with chelex-100) (Brown et al., 2017). *In vitro* biofilm formation ability of the bacteria, in the presence of ASD, was evaluated using a static microtiter plate assay (Christensen et al., 1982; Yadav et al., 2018). Viable bacteria in the biofilms were detected by colony-forming unit (cfu) counting, and metabolically active bacteria were detected by resazurin staining. Briefly, *S. pneumoniae* colonies grown on BAP were further grown in BHI broth up to log phase. The cells were pelleted by centrifugation and dissolved in metal ion-free medium. The diluted bacteria (1:100) in metal ion-free medium were inoculated in 96-well (200 μl) or 24-well (1 ml) plates and incubated for 48 h. After incubation, the medium was removed, and biofilms were washed twice and stained with 0.1% crystal violet for 15 min. The biofilms were washed with PBS twice and dissolved in 200 μl (96-well plate) or 1 ml (24-well plate) ethanol, and the absorbance was measured at 570 nm. Alternately, after washing with PBS, the biofilms were dissolved in sterile water followed by brief sonication (10 s). The biofilm suspension was serially diluted and spread on BAP, followed by colony counting after 24 h incubation at 37°C.

The *in vitro* biofilms of *Pseudomonas aeruginosa*, MRSA, and *E. coli* were grown as described above and quantified using resazurin staining (Yadav et al., 2017). Resazurin stain is a blue colored, non-fluorescent dye that is reduced and then emits pink fluorescence (resorufin) in the presence of actively growing bacteria. The biofilms grown in 24-well plates were dissolved and transferred to 96-well plates, followed by addition of 10% resazurin. The plates were incubated in the dark for 1 h at 37°C. Fluorescence was measured at 530/590 (excitation/emission) nm using a multimode microplate reader (Thermo Scientific, Waltham, MA, United States).

### *In vitro* Biofilm Analysis by Scanning Electron Microscopy (SEM)

*In vitro* biofilms of *S. pneumoniae* D39, grown in the absence or presence of ASD (300 μg/ml), were visualized using SEM. The biofilms were grown in 24-well plates, in the metal ion-free medium for 48 h using the procedure described above. After washing with PBS, the biofilms were pre-fixed in glutaraldehyde (2%) and paraformaldehyde (2.5%) and post-fixed with osmic acid (1%) for 2 h, followed by dehydration in increasing concentrations of ethanol (60–95%). The biofilms were preserved in t-butanol and freeze-dried and platinum-coated. SEM images were captured using a field emission scanning electron microscope (Hitachi, Tokyo, Japan).

### *In vitro* Biofilm Analysis by Confocal Microscopy

*Streptococcus pneumoniae* D39 *in vitro* biofilms, grown in the presence (300 μg/ml) and absence of ASD, were analyzed using confocal microscopy and peptide nucleic acid (PNA) fluorescence *in situ* hybridization (FISH) by a previously reported procedure (Malic et al., 2009). The PNA probe used for biofilm detection is a universal bacterial probe EUB338 (5′-TGCCTCCCGTAGGA-3′) (Rocha et al., 2018). It was commercially synthesized by Panagene (Dageon, South Korea) and labeled at the N-terminus with AlexaFluor488 via a double 8-amino-3,6-dioxaoctanoic acid (AEEA) linker. The biofilms were grown on μ-slides (ibidi, Germany) for 48 h in metal ion-free medium, as described above. The biofilms were washed with PBS and prefixed for 3 h in 4% paraformaldehyde. Hybridization with the probe was performed at 46°C for 3 h in hybridization buffer (5 M NaCl, 1 M Tris-HCl, 2% SDS, and 10% formamide), followed by washing at 48°C in washing buffer (5 M NaCl, 1 M Tris–HCl, and 2% SDS). The PNA probe-labeled biofilm bacteria were analyzed using a Nikon A1 confocal microscope (Nikon Instruments, Inc., NY, United States) with FITC (green) channel.

### *In vitro S. pneumoniae* Biofilm Gene Expression

Real-time RT-PCR was used to evaluate the expression of genes involved in competence (*comA*, *comB*, *ciaR*), biofilm formation (*luxS*), and toxin production (*ply, lytA*) in a pneumococcal biofilm. Pneumococcal biofilms were grown in metal ion-free medium, and total RNA was extracted using an RNeasy Total RNA Isolation System Kit (Qiagen, Valencia, CA, United States) as per the manufacturer’s procedure. DNA contamination was removed by on-column RNase-free DNase (Qiagen) treatment for 10 min at 20–25°C. The RNA was quantified using Nano-drop, and cDNA synthesis was performed using a Bioneer cDNA synthesis kit (Seoul, South Korea). Gene list and primer sequences used in this study are presented in Table 1. Real-time RT-PCR was performed in a 20-μl reaction volume with 10 μl of SYBR Green, 2-pmol primers, and 2 μl of cDNA. The PCR was performed for 40 cycles with initial denaturation at 56°C for 2 min, followed by 40 cycles of denaturation at 95°C for 30 s and annealing and extension at 60°C for 1 min. Relative quantification of gene expression was performed using the 2^–ΔΔCT^ method; a biofilm without ASD was used as the standard and 16S RNA genes as reference.

**TABLE 1:** List of primers used in real-time RT-PCR gene expression study.

**Serial no.**	**Gene name**	**Primer sequence**	**Amplicon**
1	*16s*	5′-AACCAAGTAACTTTGAAAGAAGAC-3′	126 bp
		5′-AAATTTAGAATCGTGGAATTTTT-3′	
2	*luxS*	5′-ACATCATCTCCAATTATGATATTC-3′	257 bp
		5′-GACATCTTCCCAAGTAGTAGTTTC-3′	
3	*comA*	GAGACGCGAGCCATTAAGG	156 bp
		GGGATCTGGATCGGCAATATGA	
4	*comB*	5′-GAACCCAGTCGTATCCTTGC-3′	95 bp
		5′-TCCCCCTTCTTAACCAGCTT-3′	
5	*ciaR*	GATGTTATGCAGGTATTTGATG	157 bp
		TAATCAGAACTGGTGTCGTAAT	
6	*ply*	TGAGACTAAGGTTACAGCTTACAG	225 bp
		CTAATTTTGACAGAGAGATTACGA	
7	*lytA*	CGTCCCAGGCACCATTATCA	95 bp
		CTGGCGGAAAGACCCAGAAT	

### HMEEC Viability

The toxicity of *S. pneumoniae* in the presence or absence of ASD was detected by evaluating HMEEC viability upon treatment with *S. pneumoniae* or ASD, or co-treatment with both. HMEECs were kindly provided by Dr. Lim (House Ear Institutes, Los Angeles, CA, United States) (Chun et al., 2002). HMEECs were cultured (1 × 10^4^) in a 96-well plate in airway epithelial cell growth medium (PromoCell GmbH, Sickingenstr Heidelberg Germany) supplemented with bovine pituitary extract (0.004 ml/ml), epidermal growth factor (10 ng/ml), insulin (5 μg/ml), hydrocortisone (0.5 μg/ml), epinephrine (0.5 μg/ml), triiodo-L-thyronine (6.7 ng/ml), transferrin (10 μg/ml), retinoic acid (0.1 ng/ml), and 1% fetal bovine serum for 24 h at 37°C in 5% CO_2_. Then, cells were exposed to ASD (300 μg/ml) in serum-free medium for 8 h, followed by *S. pneumoniae* treatment (MOI 10) for 15 h. The viability of HMEECs was determined by using EZ-cytox cell viability kit (Dogenbio, South Korea) as per the manufacturer’s instruction, and absorbance was measured at 450 nm.

### Detection of Apoptosis in HMEECs

Apoptosis of HMEECs treated with ASD or *S. pneumoniae* or co-treatment was detected by double staining with annexin V-fluorescein isothiocyanate and propidium iodide (BD, San Diego, CA, United States) and cytometric analysis as per manufacturer’s protocol. Briefly, HMEECs (5 × 10^5^/well) were seeded in a 6-well plate in airway epithelium cell culture medium supplemented with fetal bovine serum (1%) in 5% CO_2_ atmosphere at 37°C for 24 h. Then, the HMEECs were exposed to ASD (300 μg/ml) in serum-free medium for 8 h, followed by *S. pneumoniae* treatment (MOI 10) for 15 h. The HMEECs were detached with Tris–EDTA treatment, pelleted by centrifugation, and washed twice with cold PBS. The cells were re-suspended in 1 × binding buffer [10 mM HEPES/NaOH (pH 7.4), 140 mM NaCl, and 2.5 mM CaCl_2_] and stained with Annexin-V for 15 min at 15°C. The cells were stained with PI and evaluated using a flow cytometer (Beckman Coulter; Fullerton, CA, United States). The rates of early apoptosis and late apoptosis (necrosis) were calculated using the Beckman Coulter software.

### Detection of Reactive Oxygen Species (ROS) in HMEEC

The effect of ASD and *S. pneumoniae* on ROS production by HMEECs was evaluated using OciSelect ROS assay kit (Cell Biolabs; San Diego, CA, United States). 5 × 10^4^ HMEECs were seeded in 96-well plate (black wall clear-bottom plate) in airway epithelial growth medium and grown at 37°C in 5% CO_2_ for 24 h. After washing, the cells were treated with 100 μl 2′,7′-dichlorofluorescein-diacetate (DCFH-DA) in the culture medium at 37°C for 50 min. After washing twice with PBS, HMEECs were exposed to ASD (300 μg/ml) for 2 h, followed by *S. pneumoniae* treatment for 4 h. The ROS production was detected by measuring fluorescence at 480 nm (excitation) and 530 nm (emission) using microplate (Thermo max 190, US). Hydrogen peroxide was used as a positive control.

### *In vivo* Colonization of *S. pneumoniae* in Rat Middle Ear in the Presence of ASD Using the Rat OM Model

The *in vivo* colonization of *S. pneumoniae* in rat middle ear in the presence of ASD was evaluated using the rat OM model (Yadav et al., 2012, 2018). Thirty-two pathogen-free Sprague-Dawley rats, weighing 150–200 g, were purchased from Koatech (Pyeongtaek, South Korea). The animals were checked for any abnormality and kept for acclimatization for 2 weeks. Rats were divided into four groups according to the treatment. Group 1 rats were inoculated with PBS (vehicle control, *n* = 6); Group 2 rats were inoculated with ASD only (*n* = 8); Group 3 rats were inoculated with *S. pneumoniae* only (*n* = 9); Group 4 rats were inoculated with ASD + *S. pneumoniae* (*n* = 9). The ASD was dissolved in PBS, and 50 μl (6 mg/ml) solution was injected (300 μg/rat) as per our previous study (Chang et al., 2016). *S. pneumoniae* cell suspensions were prepared in PBS, and 5 × 10^6^/rat were injected in the middle ear cavity through the tympanic membrane of the right ear using a tuberculin syringe and a 27-gauge spine needle. After 5 days, the animals were scarified by CO_2_ euthanasia. The rat bullae were aseptically acquired and cleaned by removing unwanted tissue surrounding the bony structure. The tympanic membranes were removed, and the middle ear was exposed and photographed. For gene expression study, bullae were harvested in RNA-later solution (Qiagen, United States). For bacterial load detection, bullae were aseptically lysed with pestle and mortar and serially diluted and plated on BAP. Bacterial cfu were counted after overnight incubation at 37°C.

For SEM analysis of rat middle mucosa, the bullae from each group were preserved in SEM solution (glutaraldehyde and paraformaldehyde). The rest of the protocol (pre-fixing, post-fixing, and dehydration) was the same as that described previously for *in vitro* biofilms SEM analysis.

### Elucidation of Rat Middle Ear Mucosa Global Gene Expression Using RNA Sequencing

The mucosal membranes from bullae were recovered by scraping and three rat mucosa were pooled as one sample. Total RNA was isolated using a Qiagen RNeasy kit (Qiagen, Hilden, Germany) in accordance with the manufacturer’s instructions. The RNA was quantified using a Nanodrop, and the RNA quality was assessed by analyzing the rRNA band integrity using the Agilent RNA 6000 Nano kit (Agilent Technologies, Palo Alto, CA, United States). The gene expressions of rat middle ear mucosa inoculated with ASD or bacteria or co-treatment were analyzed by QuantSeq 3′mRNA sequencing. RNA samples were processed and library was constructed using QuantSeq 3′mRNA–Seq library prep kit (Lexogen, Inc., Austria) as per the manufacturer’s instructions. Total RNA (500 ng) from each sample was used with an oligo-dt primer containing an illumine-compatible sequence at its 5′ end and hybridized with the RNA followed by reverse transcription reaction. The RNA templates were digested and the synthesis of the second strand was initiated by a random primer containing an illumine-compatible linker sequence at its 5′ end. The library of double-stranded RNA was purified from reaction components using magnetic beads. The prepared library was amplified and added with the complete adapter sequences required for cluster generation. The finally finished library was purified from PCR components of reaction. The high-throughput sequencing was performed as single-end 75 sequencing using Next Seq 500 (Illumina, Inc., United States).

Bowtie2 program was used to align the QuantSeq 3′ mRNA-Seq reads (Langmead and Salzberg, 2012). Bowtie2 indices were either generated from genome assembly sequence or the representative transcript sequences for aligning to the genome assembly sequence or the representative transcript sequences for aligning to the genome and transcriptiome. Those aligned files were utilized for transcript assembling, abundance estimation, and differential gene expression detection. The differentially expressed genes were determined on the basis of unique and multiple alignments using coverage in Bedtools (Quinlan and Hall, 2010). Read count data were processed on the basis of quantile normalization method using EdgeR within R using Bioconductor (Gentleman et al., 2004). DAVID^1^ and Medline databases^2^ were used for gene classifications. The differentially expressed genes of rat middle ear mucosa inoculated with ASD or bacteria or co-treatment compared to untreated samples were analyzed, and fold change of ±2 was considered significant.

### Statistical Analyses

The *in vitro* biofilm experiments were performed in replicates and repeated to calculate the statistical significance. Data are represented as mean ± standard deviation. Two groups were compared, and the statistical significance was detected by Student’s *t* test. Three groups were compared by one-way ANOVA. The *P*-value < 0.05 was considered significant.

## Results

### Planktonic Growth and *in vitro* Biofilm Growth

**FIGURE 1:**
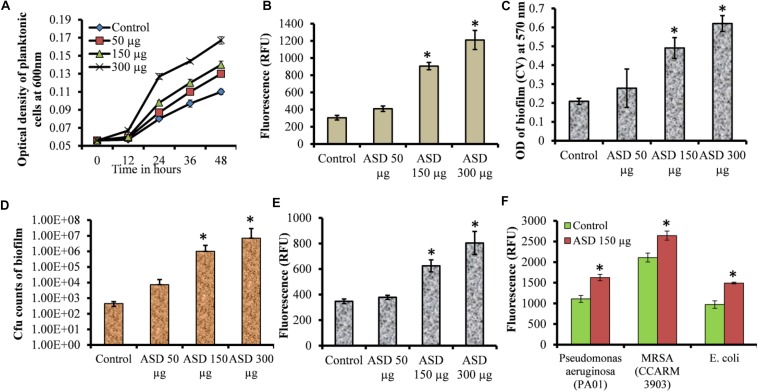
*Streptococcus pneumoniae* D39 planktonic and biofilm growth in metal ion-free BHI medium. **(A)** Time-dependent planktonic growth from 0 to 48 h with different concentrations of ASD. Growth was detected by measuring optical density at 600 nm. **(B)** Planktonic growth detected by resazurin staining after 48 h incubation. **(C)** Quantification of *in vitro* biofilm biomass grown for 48 h using a CV-microplate assay. **(D)** Colony-forming unit (cfu) counts of *in vitro* biofilms grown for 48 h. **(E)** Quantification of *in vitro* biofilm growth using resazurin staining grown for 48 h. **(F)** Quantification of *in vitro* biofilm growth of *Pseudomonas aeruginosa*, MRSA, and *E. coli* grown for 48 h using resazurin staining. Error bars are the standard deviation from the mean. Statistical significance was calculated using the Student’s *t*-test, and *P*-value < 0.05 was considered significant (^∗^*P* < 0.05).

In metal ion-free medium *S. pneumoniae* growth was restricted. However, bacterial growth was significantly elevated in the presence of ASD compared to the control (Figure 1A). The growth of bacteria was slow initially, both in the presence and in the absence (control) of ASD. However, at 24, 36, and 48 h, bacterial growth was significantly (*P* < 0.05) high in the presence of ASD (50, 150, and 300 μg/ml) compared to the control. The metabolically active bacteria were also significantly (*P* < 0.05) increased in samples supplemented with ASD (Figure 1B).

The *S. pneumoniae* D39 *in vitro* biofilm growth in the presence of ASD was enhanced compared to the control. Quantification of biofilm biomass using CV-microtiter plate assay showed significant (*P* < 0.05) increase in biofilm biomass in the presence of 150 and 300 μg/ml ASD (Figure 1C), with a significantly (*P* < 0.05) increased number of viable bacteria (Figure 1D). The metabolically active bacteria within biofilms were also significantly (*P* < 0.05) higher in the presence of 150 and 300 μg/ml ASD (Figure 1E). *Pseudomonas aeruginosa*, MRSA, and *E. coli in vitro* biofilms were also significantly (*P* > 0.05) elevated in the presence of ASD (150 μg/ml) (Figure 1F).

### *In vitro* Biofilm Analysis Using SEM

**FIGURE 2:**
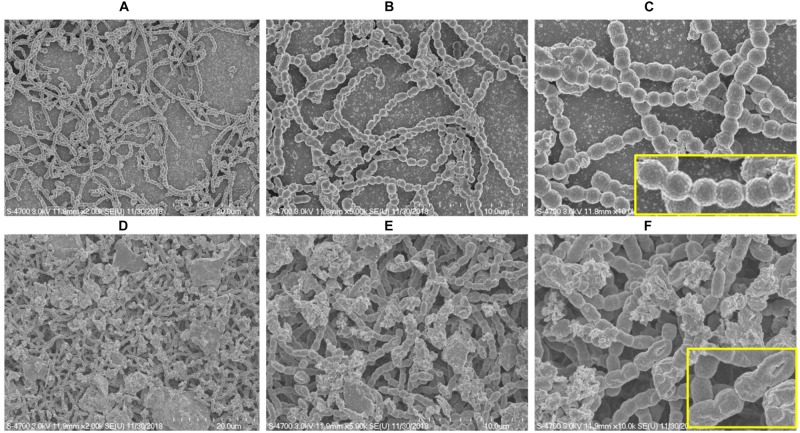
Scanning electron microscope (SEM) images of *S. pneumoniae* D39 *in vitro* biofilms grown in metal ion-free medium for 48 h. **(A–C)** Representative SEM images of the *S. pneumoniae* biofilms grown without ASD (control). The control biofilms were thin and unorganized, bacteria formed long chains, and cell size was smaller than normal. **(D–F)** SEM images of the *S. pneumoniae* biofilms grown in the presence of ASD particles (300 μg/ml). The bacteria formed compact biofilms and were attached to each other, and the cell size appeared normal. Images from left to right are 20, 10, and 5 μm, respectively.

SEM analysis revealed markedly different morphology of pneumococci *in vitro* biofilms grown with ASD (300 μg/ml) with respect to control. The biofilms grown without ASD (control) were thin, bacteria-formed long chain-like structures, and the size of bacteria appeared smaller than the normal (Figures 2A–C). In contrast, in the presence of ASD, compact and thick biofilms were formed, and the bacteria were connected to each other and to the adjacent bacteria, imbedded in particulate matters (Figures 2D–F). The size of the bacteria appeared normal in the presence of ASD.

### *In vitro* Biofilm Analysis Using Confocal Microscopy

**FIGURE 3:**
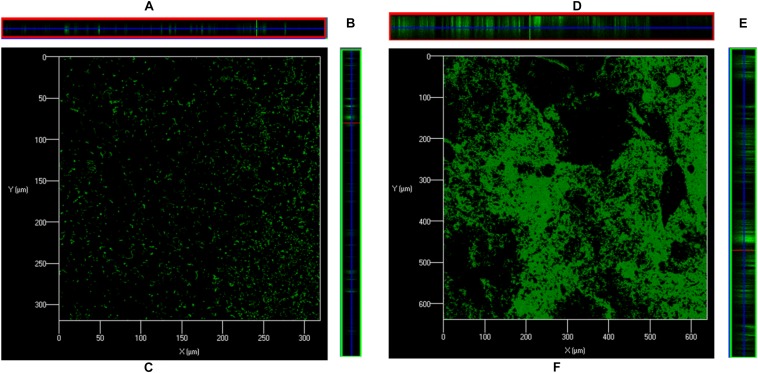
Confocal microscopy images of *S. pneumoniae* D39 *in vitro* biofilms grown in metal ion-free medium for 48 h. **(A)** Is *XZ* and **(B)** is *YZ* plane, and **(C)** is 3-D confocal microscopy image of the *S. pneumoniae* biofilm grown without ASD (control). **(D)** Is *XZ* and **(E)** is *YZ* plane, and **(F)** is 3-D confocal microscopy image of the *S. pneumoniae* biofilm grown with ASD (300 μg/ml).

The structures of biofilms, grown in the absence and presence of ASD (300 μg/ml), were analyzed by confocal microscope. The bacteria labeled with fluorescent green PNA probe were visualized. In control samples, bacteria were attached to the bottom of the plate and were unable to form organized biofilms (Figures 3A–C). In samples supplemented with ASD (300 μg/ml), cells were connected to each other and to the bottom of the plate and formed biofilms of significant depth (Figures 3D,E), and the bacteria formed three-dimensional organized biofilms (Figure 3F).

### *S. pneumoniae* Biofilm Gene Expressions Altered by ASD Presence

**FIGURE 4:**
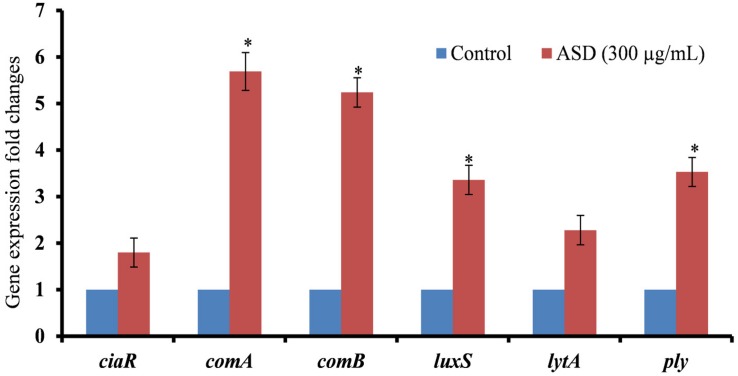
Fold changes in the gene expression of *S. pneumoniae* D39 biofilm grown in metal ion-free medium supplied with 300 μg/ml ASD for 48 h. The differential gene expression was detected by real-time RT-PCR. The error bars representing standard deviation from mean, and statistical significance were calculated by Student’s *t*-test and ^∗^*P*-value less than 0.05 was considered significant.

Our study showed that pneumococcal *in vitro* biofilm growth was enhanced in the presence of ASD; therefore, to evaluate the underlying molecular mechanism, we analyzed the expressions of genes involved in competence (*ciaR)*, competence release *(comA, comB*), and biofilm formation (*luxS*) and toxin-related (*lytA*, *ply*) genes using real-time RT-PCR. The gene expression study revealed up-regulation of genes, such as *ciaR*, *comA, comB, luxS*, *lytA*, and *ply* in biofilms grown in the presence of 300 μg/ml ASD (Figure 4). The three genes, *ciaR*, *comA*, and *comB*, involved in *S. pneumoniae* competence production and trans-membrane release of competence stimulating peptides (CSP-1) were up-regulated by 1.8, 5.6, and 5.2-fold, respectively. The *luxS* gene, involved in autoinducer-2 production, a quorum-sensing molecular system, was up-regulated in the presence of ASD by 3.4-fold. Similarly, the pneumococcal toxin-related genes, *ply* (3.5-fold) and *lytA* (2.3-fold), were up-regulated in the presence of ASD.

### ASD Exposure Decreased HMEECs Viability and Increased Apoptosis

**FIGURE 5:**
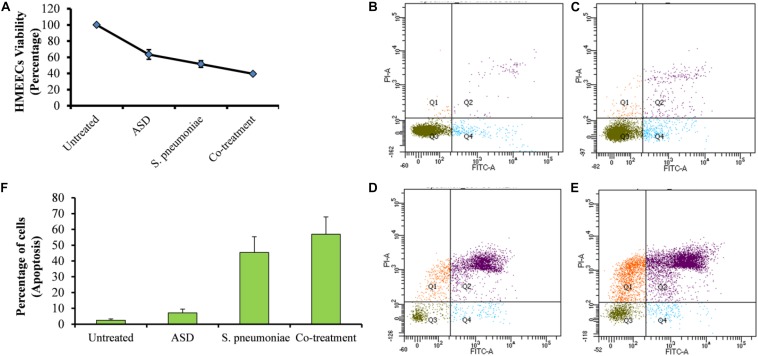
The viability of HMEECs and apoptosis upon ASD or *S. pneumoniae* treatment or co-treatment. **(A)** Cell viability results are represented as the percentage of viable cells compared to the untreated cells (100%). The error bars represent the standard deviation. **(B)** Apoptosis of HMEECs in control, **(C)** apoptosis of HMEECs upon ASD treatment, **(D)** apoptosis upon *S. pneumoniae* treatment, and **(E)** apoptosis of HMEECs upon co-treatment, detected by annexin-V/PI double staining and cytometry analysis. **(F)** Percentage of HMEECs undergoing early and late apoptosis.

Percent decrease in cell viability is shown in Figure 5. Viability of the untreated cells (control) was considered 100%, whereas that of treated cells was calculated. HMEECs viability was approximately 63% upon ASD treatment or 51% on *S. pneumoniae* treatment; however, on co-treatment (ASD + *S. pneumoniae*), the cell viability was 39% (Figure 5A). In co-treatment, the HMEECs viability was significantly (*P* < 0.05) decreased by 60% (Figure 5A).

The apoptosis of HMEECs was detected by annexin-V/PI double staining and cytometry analysis. The cytometric analysis showed that upon single treatment of HMEECs with ASD (Figure 5C) or *S. pneumoniae* (Figure 5D), a lower number of cells undergo apoptosis. However, large cells undergo apoptosis on co-treatment (Figure 5F). The percentage of cells undergoing apoptosis with co-treatment was markedly increased compared to that with ASD or *S. pneumoniae* single treatment (Figure 5F).

### ASD Exposure Caused Elevated ROS Production

**FIGURE 6:**
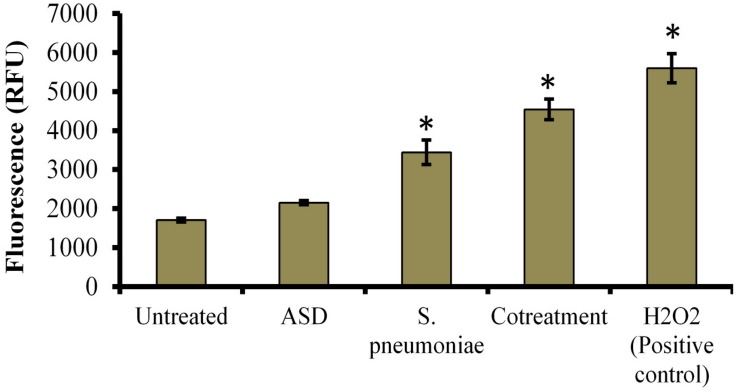
Reactive oxygen species (ROS) production in HMEECs treated with *S. pneumoniae* or ASD or co-treatment. The error bars represent the standard deviation from the mean, and statistical significance was calculated by one-way ANOVA. ^∗^*P*-value less than 0.05 was considered significant.

Treatment of HMEECs with ASD or *S. pneumoniae* causes toxicity; one of the mechanisms of toxicity is ROS production. Here, we measured ROS production in HMEECs exposed to either ASD or *S. pneumoniae* or co-treatment. ROS production was increased in HMEECs upon single treatment with ASD or *S. pneumoniae*. However, the ROS production was significantly (*P* < 0.05) elevated on co-treatment (Figure 6). Thus, ROS production on co-treatment can be attributed to both ASD and *S. pneumoniae*.

### ASD and *S. pneumoniae* Co-treatment Increased Bacterial Colonization in Rat Middle Ear Mucosa

**FIGURE 7:**
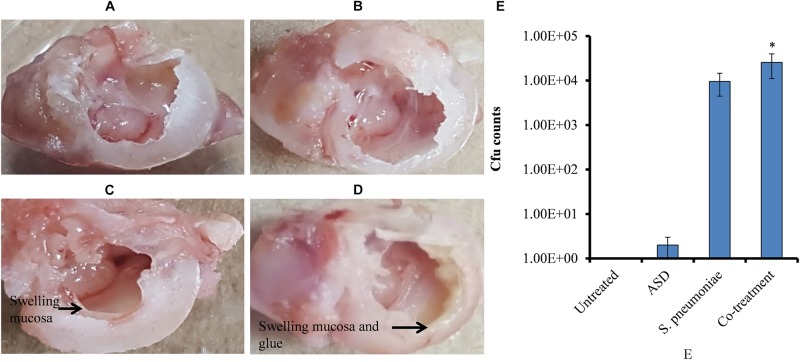
**(A–D)** Digital photograph of rat middle ear injected with ASD or *S. pneumoniae* of co-treatment. **(E)** Cfu counts of rat middle ear inoculated with ASD or *S. pneumoniae* or co-treatment. The error bars representing standard deviation from mean, and statistical significance were calculated by one-way ANOVA and ^∗^*P* value less than 0.05 was considered significant.

*In vivo* study showed no visible middle ear mucosal swelling in the control rat bulla (Figure 7A), although swelling of middle ear mucosa was visible in ASD (Figure 7B) or *S. pneumoniae* (Figure 7C) or co-treatment (Figure 7D). In co-treatment, severe swelling of the mucosa, with glue-like deposition, was visible. *In vivo* colonization of *S. pneumoniae* in the presence of ASD was increased. The cfu counts of the rat middle ear injected with ASD + *S. pneumoniae* showed increased bacteria colonization. In co-treatment, significantly (*P* < 0.05) > 65% more cfu counts were detected compared to the bacteria-only treatment (Figure 7E).

**FIGURE 8:**
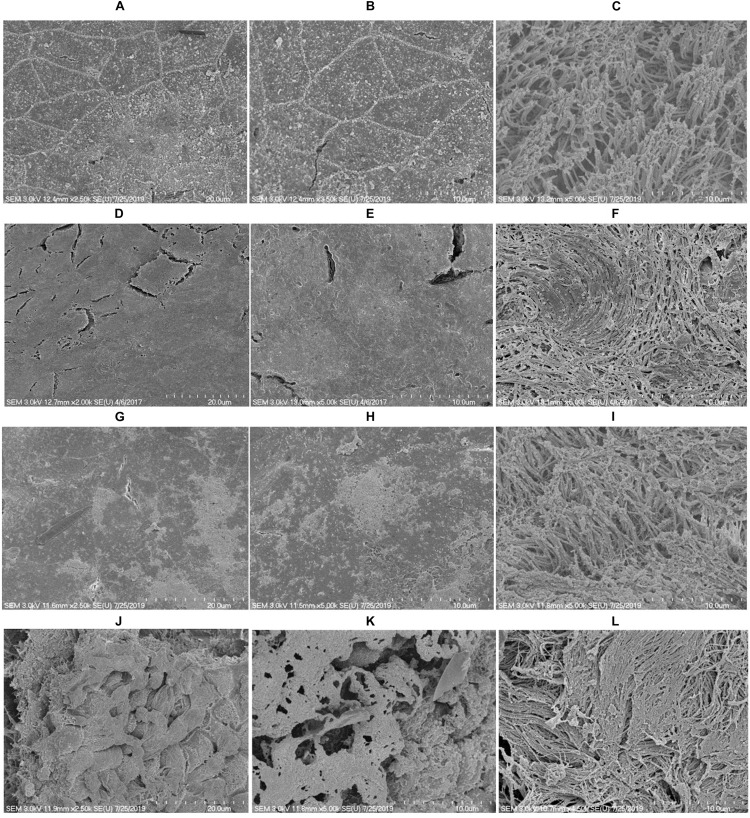
SEM images of rat middle ear colonized with *S. pneumoniae* D39 in the presence or absence of ASD. **(A–C)** Are SEM images of rat middle ear of vehicle control. **(D–F)** Are SEM images of rat middle ear injected with ASD (300 μg/ml). **(G–I)** Are SEM images of rat middle ear infected with *S. pneumoniae* only. **(J–L)** Are SEM images of rat middle ear injected with ASD + *S. pneumoniae*.

To evaluate the alteration in rat middle ear mucosa morphology upon ASD or *S. pneumoniae* colonization, SEM analysis was conducted. The rat middle ear mucosa is composed of ciliated epithelium in the hypo-tympanic area and eustachian tube orifice area, and the remaining middle ear is covered with non-ciliated squamous epithelium. The SEM analysis showed a clean middle ear of the control rat (Figures 8A,B) with visible cilia (Figure 8C). The rat middle ear injected with ASD only was clean in the non-ciliated area (Figures 8D,E); however, the cilia of the ciliated epithelium were conglomerated (Figure 8F). The rat middle ear infected with *S. pneumoniae* showed some biofilm-like debris deposition on the non-ciliated epithelium (Figures 8G,H), and the cilia of the ciliated epithelium were conglomerated (Figure 8I). Interestingly, the SEM analysis of the rat middle ear injected with ASD+ *S. pneumoniae* revealed that the non-ciliated squamous epithelium was completely filled with biofilm-like debris that covered the whole middle ear (Figures 8J,K). The cilia of the ciliated epithelium were conglomerated, and debris was deposited on the tips of cilia (Figure 8L).

### Elucidation of Rat Middle Ear Mucosa Global Gene Expression Using RNA Sequencing

**FIGURE 9:**
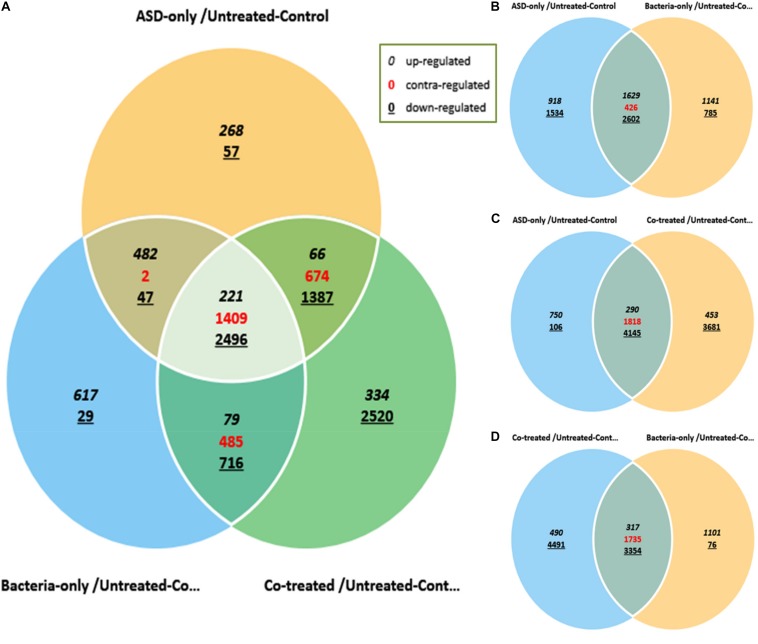
Venn diagram showing differentially expressed genes of rat middle ear mucosa inoculated with ASD only or *S. pneumoniae* (bacteria) or co-treatment with respect to untreated. The global gene expressions were evaluated by Quant 3′m-RNA Sequencing. **(A)** Differentially expressed genes by ±2-folds in all three treatments with respect to control. **(B)** Venn diagram showing differential gene expressions in ASD or *S. pneumoniae* treatment. **(C)** Venn diagram showing differential gene expressions in ASD or co-treatment. **(D)** Venn diagram showing differential gene expressions in *S. pneumoniae* or co-treatment.

The differential gene expressions of rat middle ear mucosa inoculated with ASD or *S. pneumoniae* or co-treatment were analyzed by Quant 3′mRNA sequencing. The differentially gene expression analysis revealed a total of 7109 genes that were differentially regulated in ASD-only treatment with respect to untreated. In bacteria-only treatment, 6583 genes were differentially regulated, while the total number of genes differentially expressed in co-treatment were 10,387 (Figures 9A–D).

**FIGURE 10:**
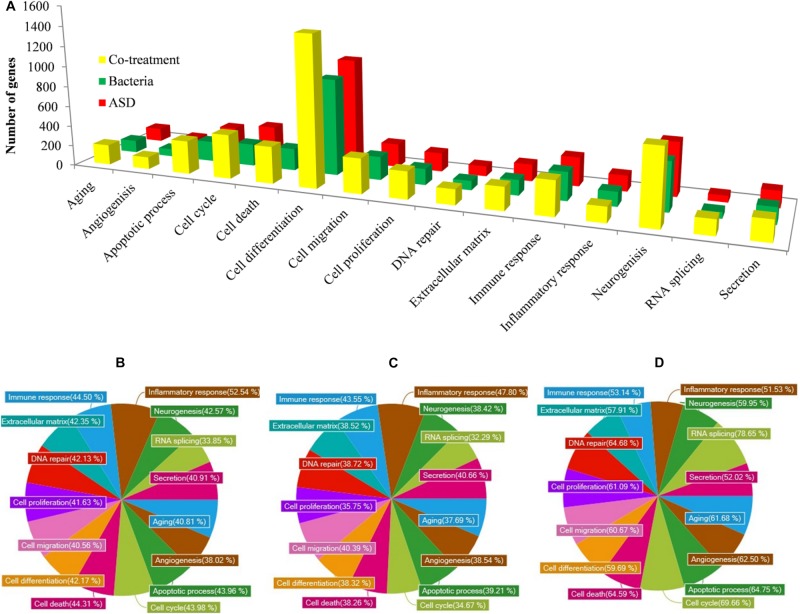
GO (Biological term) of genes differentially expressed in rat middle ear mucosa treated with ASD or *Streptococcus pneumoniae* or co-treatment with respect to control (untreated). **(A)** Number of gene and functional categories. **(B)** Percentage of significantly differentially expressed genes involved in different functional categories and up-regulated or down-regulated upon ASD treatment, (B) *S. pneumoniae* treatment, or **(C)** co-treatment.

The gene ontology (GO) analysis revealed that the genes involved in immune response, inflammatory response, DNA repair, cell cycle, cell death, apoptosis process, etc. were differentially expressed in all three treatments (Figure 10A). However, the number of genes differentially expressed in the above categories were higher in co-treatment compared to single treatments. For example, immune response-related genes differentially expressed in co-treatment were 338, while those in ASD or *S. pneumoniae* treatment were 283 and 277, respectively. Similarly, in co-treatment, 1472 genes related to cell differentiation were differentially regulated, while in ASD or *S. pneumoniae*, 1040 and 945 genes, respectively. The cell death-related genes differentially expressed in co-treatment or ASD or *S. pneumoniae* were 363, 249, and 216, respectively. Apoptosis-related genes differentially regulated in co-treatment or ASD or *S. pneumoniae* treatment were 327, 222, and 198 respectively. These results indicate that the co-treatment induces a large number of gene expressions and affects a large number of cellular processes.

The percentages of significantly differentially regulated genes and the functional category are shown in Figure 10. In ASD treatment, the percentage of differentially expressed genes involved in immune response, inflammatory response, DNA repair, cell cycle, cell death, and apoptosis process was 44.50, 52.54, 42.13, 43.98, and 44.31%, respectively (Figure 10A). In *S. pneumoniae* treatment, the percentage of differentially expressed genes involved in immune response, inflammatory response, DNA repair, cell cycle, cell death, and apoptosis process was 43.55, 47.80, 38.72, 34.67, 38.26, and 39.21%, respectively (Figure 10B). However, in co-treatment, the percentage of genes that were differentially regulated in each of the above categories was significantly higher. In co-treatment, the percentage of differentially expressed genes involved in immune response, inflammatory response, DNA repair, cell cycle, cell death, and apoptosis process genes were 53.14, 51.53, 64.68, 69.66, 64.59, and 64.75%, respectively (Figure 10C). The significantly expressed genes involved in apoptosis, cell death, immune response, and inflammatory response and fold change in three treatments are given in the Supplementary Table.

OM-related genes differentially regulated in three treatments include lysine 63 deubiquitinase encoding gene (CYLD), the heme oxygenase 1 encoding gene (HMOX1), the surfactant protein D encoding gene (SFTPD), the SMAD family member 4 encoding gene (SMAD4), the F-Box protein 11 encoding (FBXO11), CD14 molecule encoding gene (CD14), tumor necrosis factor (TNF), Interleukin 1 beta encoding gene (IL1B), and Interleukin 1 alfa encoding (IL1a). In addition, the antibacterial peptide/protein encoding gene such as NP4 (encodes defensin NP-4 precursor), seven genes encoding defensin (DEFB1, DEFA5, DEFA7, DEFA8, DEFA10, DEFA11, and RATNP-3B), CTSG (encode cathepsin G), and six genes of S100 family (encoding S100 calcium binding protein) were down-regulated in co-treatment. Those genes were non-significantly (<2-fold) down-regulated or down-regulated by less folds compared to co-treatment (Table 2).

**TABLE 2:** Otitis media-related differentially expressed genes in rat mucosa treated with ASD or *Streptococcus pneumoniae* or co-treatment.

**Gene function**	**Gene name**	**Fold change in ASD**	**Fold change in *Streptococcus pneumoniae***	**Fold change co-treatment**
Host defense against microbial infection	CTSG gene encodes cathepsin G transcript variant X1	0.05	0.07	0.03
	S100a13 gene encodes S100 calcium binding protein A13	0.45	0.48	0.003
	S100a16 gene encodes S100 calcium binding protein A16	1.5	1.5	0.002
	S100a3 gene encodes S100 calcium binding protein A3, transcript variant X2	0.21	0.51	0.21
	S100a8 gene encodes S100 calcium binding protein A8, transcript variant X1	0.42	0.37	0.042
	S100a9 gene encodes S100 calcium binding protein A9, transcript variant X1	0.51	0.26	0.04
	NP-4 gene encodes defensin NP-4 precursor	0.062	0.08	0.03
	DEFB1 gene encodes defensin beta 1	1.7	8.0	0.25
	DEFA5 gene encodes defensin alpha 5, transcript variant X1	0.042	0.01	0.003
	DEFA7 gene encodes defensin alpha 7	0.080	0.090	0.04
	DEFA10 gene encodes defensin alpha 10	0.41	0.38	0.31
	DEFA11 gene encodes defensin alpha 11, transcript variant X1	0.05	0.09	0.07
	RATNP-3B gene encodes defensin RatNP-3 precursor	0.12	0.2	0.07
Inflammatory response	SMAD4 gene encodes SMAD family member 4	1.471	1.439	0.02
	HMOX1 gene encodes the heme oxygenase 1 encoding gene	9.578	22.228	14.609
	CYLD gene encodes deubiquitinase cylindromatosis	Cyld	1.686	1.204
	FBXO11 gene encodes the F-Box protein 11 encoding	2.304	1.198	0.006
	CD14 gene encodes molecule encoding gene	11.958	6.473	12.230
Cytokines and interleukins	TNF gene encodes tumor necrosis factor	117.15	60.40	0.95
	IL1B gene encodes Interleukin 1 beta encoding gene	4257.707	1057.356	788.250
	IL1A gene encodes Interleukin 1 alfa encoding	397.876	98.273	0.953
Apoptosis	Ddit3 encodes DNA-damage inducible transcript 3, transcript variant X2	2.066	3.676	3.846
	Bak1 gene encodes BCL2-antagonist/killer 1, transcript variant X1	2.592	2.314	2.503

## Discussion

*Streptococcus pneumoniae* and ASD particulate matter are among the major risk factors causing OM worldwide; however, these two factors have been studied separately until now (Hall-Stoodley et al., 2006; Park et al., 2018). Attempts have not been made to evaluate the synergistic or additive effects of these risk factors on the outcome of OM. Furthermore, the initial interaction of ASD and pneumococci occurs in the nasopharyngeal cavity, and *S. pneumoniae* is a commensal bacterium that colonizes the nasopharyngeal cavity asymptomatically. It is not known whether the pneumococci revert to the pathogenic form on exposure to particulate matter such as ASD and cause OM. In this study, we evaluated the effect of ASD on pneumococcal biofilm growth and colonization on HMEECs and on middle ear mucosa using the rat OM model.

In this study, our results showed low bacterial growth in metal ion-free medium; however, in the presence of ASD, pneumococcal growth was significantly increased, indicating that ASD composition plays a crucial role in pneumococcal growth in the metal-devoid medium. For normal bacterial growth, metals such as Fe, Na, Mg, and Mn are essential; however, those were absent in metal ion-free medium (Weiss and Carver, 2018). Previously, Hussey et al. (2017) using Todd–Hewitt broth + 0.5% (w/v) yeast extract (THY), which contains all the required essential elements detected increased bacterial growth in the presence of black carbon. However, no reasons for elevated bacterial growth were suggested (Hussey et al., 2017). We previously reported that ASD contains various metals such as Fe, Na, Mg, and Mn (Chang et al., 2016). Since Fe, Mg, and Mn are important for *S. pneumoniae* growth and virulence, bacterial growth and virulence were disrupted in the absence of those metals (Romero-Espejel et al., 2013; Weiser et al., 2018). Pneumococci possess a specific efflux pump for the utilization of Fe and other metals (Honsa et al., 2013). Therefore, it appears that the metal contents of ASD favor pneumococcal planktonic growth.

We detected increased *in vitro* biofilm growth in the presence of ASD. The primary reason for elevated biofilm growth of pneumococci may be the increase in planktonic bacterial growth. In addition, the metal contents of ASD, including Fe (2.035%) stimulated biofilm growth. An increase in biofilm growth and virulence of pneumococci in the presence of Fe has been reported previously (Trappetti et al., 2011b). The biofilms were grown in metal-free medium, and SEM analysis revealed a significant morphological difference in the control and ASD-treated biofilms. In the absence of metal ion, pneumococci cell size appeared abnormal and formed long chain-like structures. Iron is an essential metal for bacterial growth, and its importance in pneumococcal growth is well-known (Romero-Espejel et al., 2013). In contrast, the biofilms supplemented with ASD were thick and compact and formed 3-D structures, and the bacteria size appeared normal (Moscoso et al., 2006), suggesting that metal deficiency was compensated for by the metal constituents of ASD, and the pneumococci resumed normal biofilm growth. Similarly, in the presence of Fe, up-regulation of biofilm formation has been reported for *Pseudomonas* and *E. coli* (Banin et al., 2008; Wu and Outten, 2009). Probably, the ASD particles provide a favorable surface for bacterial attachment and biofilm growth. Similar results were observed previously in the presence of black carbon (Hussey et al., 2017).

In *S. pneumoniae*, the production of biofilms was found to be regulated by competence (Com) quorum-sensing (QS) mediated by the competence-stimulating peptide (CSP) and LuxS/Autoinducer-2 (AI-2) QS (Trappetti et al., 2011a; Vidal et al., 2013; Weyder et al., 2018). In this study, up-regulation of *ciaR*, *comA*, and *comB* and *luxS* gene indicated that competence and QS were increased in the presence of ASD. Trappetti et al. (2011b) reported that *luxS* gene is the central regulator of competence, fratricide, and biofilm formation, and its expression is up-regulated in the presence of Fe in pneumococci (Trappetti et al., 2011b). In addition, the *luxS* gene is important for the synthesis of the AI-2 QS molecule that regulates pneumococcal biofilms; less biofilm formation has been reported in the *S. pneumoniae luxS* mutant (Vidal et al., 2013; Yadav et al., 2018). The *ply* genes encoding pneumolysin and *lytA* encode protein that facilitates the release of toxin that was up-regulated in biofilms (Allegrucci et al., 2006; Moscoso et al., 2006). Altogether, these results indicated that ASD induced the expression of biofilm and competence-related genes and enhanced biofilm formation.

Exposure to ASD is toxic to epithelial cells; our previous study showed a concentration-dependent decrease in HMEEC viability in the presence of ASD (Chang et al., 2016). Exposure to pneumococci also decreases the epithelial cell viability. Here, the HMEEC viability was significantly reduced in co-treatment with ASD+ *S. pneumoniae*. The significantly low viability of HMEECs in co-treatment could be due to the pre-exposure to ASD-induced inflammation, which might result in HMMEC injury, and therefore, cells become more susceptible to pneumococci infection and cell death (Willemse et al., 2005). These results suggest that the low viability in co-treatment could be the result of additive toxicity of ASD and *S. pneumoniae*.

Here, we detected a large number of HMEECs that underwent apoptosis and produced elevated ROS in co-treatment compared to the single treatments. *S. pneumoniae* is known to produce a number of toxins, including pneumolysin that induces DNA damage and cell cycle arrest (Rai et al., 2016). In addition, it was suggested that *S. pneumoniae* produces hydrogen peroxide that damages the DNA and induces apoptosis in lung epithelial cells (Rai et al., 2015). Similarly, the toxicity of ASD is attributed to oxidative stress and apoptosis (Go et al., 2015; Chang et al., 2016; Pfeffer et al., 2018). Therefore, it appears that the additive effects of ASD and *S. pneumoniae* enhance apoptosis. It is known that ASD containing PM and *S. pneumoniae* treatment individually are toxic to epithelium cells, and one of the mechanisms of toxicity is mediated by ROS production (Li et al., 2015; Rai et al., 2015). It is reported that ASD stimulates the ROS production, and in lung infection, *S. pneumoniae*-mediated hydrogen peroxide production depended on the pneumococcal autolysin LytA (Hocke et al., 2014). In addition, *S. pneumoniae* induces autophagy in A549 cells through ROS hypergeneration (Li et al., 2015). Therefore, it appears that the ASD-only or *S. pneumoniae-*only treatment was unable to induce much toxicity, apoptosis, and ROS production in HMEECs, which was amplified in co-treatment due to combined effects.

*In vivo* study results showed elevated pneumococci in rat middle ear and mucosa swelling in the presence of ASD. The concentration of ASD (300 μg/ear) or the number of bacteria injected (5 × 10^6^) was decided on the basis of our previous study, and the concentrations were reduced to exert minimum effects of single treatments (Yadav et al., 2012; Go et al., 2015; Chang et al., 2016). The cfu counts and SEM analysis indicated that a single treatment with ASD (300 μg) or *S. pneumoniae* (5 × 10^6^) induced low toxicity; however, the presence of ASD+ *S. pneumoniae* amplified the toxicity and elevated colonization on middle ear mucosa. Previously, Hussey et al. (2017) also reported increased *in vivo* colonization of bacteria in the presence of black carbon PM (Hussey et al., 2017).

The gene expression results indicate that the co-treatment induces a large number of gene expressions and affects a large number of cellular processes, which could be due to elevated toxicity in co-treatment. The gene expression study revealed a large number of genes that were significantly differentially expressed in co-treatment are involved in apoptosis, cell death, DNA repair, immune response, and inflammatory response. Inflammatory cytokines such as IL1α, IL1β, and tumor necrosis factor produced by macrophages and monocytes in response to microbial toxin play a vital role in middle ear inflammation and OM (Yellon et al., 1991; Willett et al., 1998). Here, our results showed increased expressions of cytokine- and interleukin-related genes in all three treatments; however, the fold change varied in each treatment. The SMAD gene, CYLD gene (encodes deubiquitinase cylindromatosis), and FBXO11 (F-Box Protein 11) gene were down-regulated by >2-fold in co-treatment and non-significantly in ASD or *S. pneumoniae* treatment. The SMAD genes are mediators of the TGF-β pathway and regulate cell proliferation, apoptosis, and cell differentiation and mutation in SMAD-increased OM susceptibility (MacArthur et al., 2014). The FBXO11 is another important OM-related gene in mouse model mutation in FBXO11 that caused OM (Hardisty-Hughes et al., 2006). The deubiquitinase cylindromatosis (CYLD) gene expression involved in the suppression of the *H. influenzae* induced expression of pro-inflammatory chemokines (Wang et al., 2014). It has been suggested that PM weakens the host innate defense and obstructs the antibacterial peptides and proteins such as secretory leukocyte protease inhibitor and defensins (Chen et al., 2010, 2018). Here, our results showed down-regulation of NP4 (encodes defensin NP-4 precursor), CTSG (encode cathepsin G), six genes of the S100A family (S100A13, S100A16, S100A3, S100A6, S100A8, and S100A9) and seven genes encoding defensin (DEFB1, DEFA5, DEFA7, DEFA8, DEFA10, DEFA11, and RATNP-3B) in co-treatment. These genes encode peptides or proteins that are involved in host defense against bacterial infection, and the down-regulation in co-treatment indicates that the host defense was decreased. Defensins are broad-spectrum antimicrobial peptides that have been implicated in prevention of AOM (Underwood and Bakaletz, 2011). It was reported that deficiency of S100A8/A9 in mice could promote the progression of pneumonia caused by bacterial infection (Achouiti et al., 2015). The gene expression results indicates that co-treatment up-regulated pro-inflammatory cytokines and interleukins and down-regulated inflammation suppressor genes. Altogether, these results suggest that ASD exposure decreases host cell immune defense causing cells susceptible to establish pneumococcal infections and aggregate on the mucosa.

## Conclusion

The results of this study showed that in the presence of ASD, pneumococcal *in vitro* biofilm growth and *in vivo* colonization to rat middle ear mucosa were elevated. The pre-exposure to ASD increased pneumococcal colonization to HMEECs, elevated ROS production and apoptosis, and increased bacteria susceptibility results in reduced HMEEC viability. The co-treatment affects a large number of genes involved in apoptosis, cell death, immune response, inflammatory response, and down-regulated defense-related genes. Altogether, these results indicate that ASD presence decreases host immune defense and increases cell susceptibility to pneumococcal infection.

## Data Availability Statement

The raw data supporting the conclusions of this manuscript will be made available by the authors, without undue reservation, to any qualified researcher.

## Ethics Statement

The animal study was reviewed and approved by Institutional Animal Care and Use Committees (IACUCs), Korea University. Written informed consent was obtained from the owners for the participation of their animals in this study.

## Author Contributions

MY, S-WC, and J-JS: conceptualization. MY and YG: methodology. MP and J-JS: supervision. MY and J-JS: writing. MP and S-WC: review and editing.

## Conflict of Interest

The authors declare that the research was conducted in the absence of any commercial or financial relationships that could be construed as a potential conflict of interest.

## Footnotes

1 http://david.abcc.ncifcrf.gov/

2 http://www.ncbi.nlm.nih.gov/
