# Supplementary material for: Asian Sand Dust Particles Increased Pneumococcal Biofilm Formation in vitro and Colonization in Human Middle Ear Epithelial Cells and Rat Middle Ear Mucosa
Source: Front Genet. 2020 Apr 24;11:323. doi: 10.3389/fgene.2020.00323 (PMC7193691; doi:10.3389/fgene.2020.00323)
Supplement: Supplementary file 1 [file Table_1.DOCX]

**Supplementary Table -1**

Table -1: Apoptosis, cell death, immune response and inflammatory response related differentially expressed gene of rat middle ear mucosa treated with ASD or *Streptococcus pneumoniae* or co-treatment.

**Apoptosis**

| **Gene name** | **Protein** | | **ASD** | | **Streptococcus pneumoniae** | **Co-treatment** |  |
| --- | --- | --- | --- | --- | --- | --- | --- |
| Pdcd6 | programmed cell death 6 | | 3.089 | | 2.876 | 0.123 |  |
| Rps6ka2 | ribosomal protein S6 kinase polypeptide 2, transcript variant X2 | | 0.071 | | 0.288 | 0.004 |  |
| Pdcd2 | programmed cell death 2 | | 7.108 | | 6.916 | 0.003 |  |
| Tgfb1 | transforming growth factor, beta 1 | | 2.553 | | 4.035 | 24.886 |  |
| Apbb1 | amyloid beta precursor protein binding family B member 1, transcript variant X2 | | 0.251 | | 0.036 | 0.004 |  |
| Prkcb | protein kinase C, beta, transcript variant X1 | | 0.155 | | 0.204 | 0.04 |  |
| Atp2a1 | ATPase sarcoplasmic/endoplasmic reticulum Ca2+ transporting 1 | | 0.076 | | 0.065 | 0.02 |  |
| Prdx5 | peroxiredoxin 5 | | 13.007 | | 13.406 | 3.311 |  |
| Sgms1 | sphingomyelin synthase 1 | | 2.495 | | 0.475 | 0.046 |  |
| Pdcd4 | programmed cell death 4, transcript variant X5 | | 0.014 | | 0.417 | 0.014 |  |
| Cyfip2 | cytoplasmic FMR1 interacting protein 2 | | 0.344 | | 0.458 | 0.03 |  |
| Vdac1 | voltage-dependent anion channel 1 | | 0.492 | | 0.405 | 0.141 |  |
| Trpv2 | transient receptor potential cation channel, subfamily V, member 2, transcript variant 1 | | 97.846 | | 107.928 | 394.657 |  |
| Aldoc | aldolase, fructose-bisphosphate C | | 0.002 | | 0.036 | 0.20 |  |
| Pdk2 | pyruvate dehydrogenase kinase 2 | | 0.294 | | 0.271 | 0.30 |  |
| Ngfr | nerve growth factor receptor, transcript variant X1 | | 0.344 | | 0.334 | 0.046 |  |
| Mapt | microtubule-associated protein tau | | 0.142 | | 0.037 | 0.001 |  |
| Prkca | protein kinase C, alpha, transcript variant X1 | | 0.007 | | 0.037 | 0.007 |  |
| Nptx1 | neuronal pentraxin 1 | | 0.016 | | 0.016 | 0.016 |  |
| Aatk | apoptosis-associated tyrosine kinase, transcript variant X1 | | 0.144 | | 0.051 | 0.06 |  |
| Robo2 | roundabout guidance receptor 2, transcript variant X3 | | 2.423 | | 0.025 | 0.025 |  |
| Casr | calcium-sensing receptor, transcript variant X2 | | 0.043 | | 0.043 | 0.043 |  |
| Aifm3 | apoptosis inducing factor, mitochondria associated 3, transcript variant X2 | | 0.022 | | 0.022 | 0.022 |  |
| Brca2 | BRCA2, DNA repair associated, transcript variant X1 | | 0.013 | | 0.301 | 0.013 |  |
| Rnf34 | ring finger protein 34, transcript variant X2 | | 0.286 | | 0.445 | 0.07 |  |
| Taok3 | TAO kinase 3 | | 0.133 | | 0.242 | 0.05 |  |
| G0s2 | G0/G1switch 2 | | 96.375 | | 10.424 | 16.015 |  |
| Acin1 | apoptotic chromatin condensation inducer 1, transcript variant X5 | | 0.457 | | 0.411 | 0.05 |  |
| Gjb6 | gap junction protein, beta 6, transcript variant X5 | | 0.012 | | 0.331 | 0.012 |  |
| Clu | clusterin | | 9.011 | | 7.174 | 2.558 |  |
| Nkx3-1 | NK3 homeobox 1 | | 0.061 | | 0.061 | 0.061 |  |
| Tnfrsf10b | tumor necrosis factor receptor superfamily, member 10b | | 0.040 | | 0.040 | 0.040 |  |
| Slc25a4 | solute carrier family 25 member 4 | | 0.398 | | 0.405 | 0.318 |  |
| Ryr2 | ryanodine receptor 2, transcript variant 2 | | 0.020 | | 0.111 | 0.020 |  |
| Sra1 | steroid receptor RNA activator 1 | | 3.460 | | 2.608 | 2.617 |  |
| Acaa2 | acetyl-CoA acyltransferase 2 | | 0.191 | | 0.494 | 0.04 |  |
| Mt3 | metallothionein 3 | | 0.131 | | 0.307 | 0.01 |  |
| Hmox1 | heme oxygenase 1 | | 9.578 | | 22.228 | 14.609 |  |
| Tox3 | TOX high mobility group box family member 3, transcript variant X1 | | 0.013 | | 0.042 | 0.013 |  |
| Siah1 | siah E3 ubiquitin protein ligase 1 | | 0.361 | | 0.451 | 0.2 |  |
| Nol3 | nucleolar protein 3, transcript variant X1 | | 0.117 | | 0.134 | 0.12 |  |
| Zmat3 | zinc finger, matrin type 3 | | 3.026 | | 0.485 | 0.026 |  |
| Foxo1 | forkhead box O1 | | 3.565 | | 0.060 | 0.060 |  |
| Siah2 | siah E3 ubiquitin protein ligase 2 | | 2.629 | | 0.477 | 0.050 |  |
| S100a8 | S100 calcium binding protein A8, transcript variant X1 | | 0.491 | | 0.370 | 0.042 |  |
| Mllt11 | myeloid/lymphoid or mixed-lineage leukemia; translocated to, 11 | | 0.095 | | 0.131 | 0.03 |  |
| Gclm | glutamate cysteine ligase, modifier subunit | | 0.423 | | 0.079 | 0.002 |  |
| Casp6 | caspase 6, transcript variant X2 | | 0.138 | | 0.362 | 0.011 |  |
| Ddit4 | DNA-damage-inducible transcript 4 | | 15.813 | | 13.501 | 4.340 |  |
| Madd | MAP-kinase activating death domain | | 0.028 | | 0.358 | 0.028 |  |
| Apip | APAF1 interacting protein, transcript variant X1 | | 0.020 | | 0.188 | 0.020 |  |
| Rmdn3 | regulator of microtubule dynamics 3 | | 0.441 | | 0.141 | 0.010 |  |
| Il1b | interleukin 1 beta | | 4257.707 | | 1057.356 | 788.250 |  |
| Il1a | interleukin 1 alfa | | 397.876 | | 98.273 | 0.953 |  |
| Ptgis | prostaglandin I2 (prostacyclin) synthase | | 0.003 | | 0.181 | 0.003 |  |
| Snca | synuclein alpha | | 0.003 | | 0.032 | 0.003 |  |
| Mcm2 | minichromosome maintenance complex component 2 | | 0.342 | | 2.471 | 0.09 |  |
| Cidec | cell death-inducing DFFA-like effector c, transcript variant 3 | | 0.033 | | 0.033 | 0.033 |  |
| Gapdh | glyceraldehyde-3-phosphate dehydrogenase, transcript variant X1 | | 0.348 | | 0.495 | 0.03 |  |
| Epha7 | Eph receptor A7, transcript variant X1 | | 0.035 | | 0.035 | 0.035 |  |
| Xpa | XPA, DNA damage recognition and repair factor | | 0.362 | | 0.490 | 0.018 |  |
| Jun | Jun proto-oncogene, AP-1 transcription factor subunit | | 3.511 | | 2.881 | 8.080 |  |
| Faf1 | Fas associated factor 1 | | 0.156 | | 0.336 | 0.05 |  |
| Tnfrsf14 | TNF receptor superfamily member 1B | | 0.011 | | 0.046 | 0.011 |  |
| Birc6 | baculoviral IAP repeat-containing 6 | | 0.177 | | 0.382 | 0.08 |  |
| Egln3 | egl-9 family hypoxia-inducible factor 3 | | 3.043 | | 0.375 | 0.09 |  |
| Six1 | SIX homeobox 1 | | 0.104 | | 0.278 | 0.07 |  |
| Psen1 | presenilin 1, transcript variant X1 | | 0.022 | | 0.124 | 0.022 |  |
| Ddit3 | DNA-damage inducible transcript 3, transcript variant X2 | | 2.066 | | 3.676 | 3.846 |  |
| Ebag9 | estrogen receptor binding site associated, antigen, 9 | | 0.353 | | 0.455 | 0.05 |  |
| Tnfrsf11b | TNF receptor superfamily member 11B | | 0.060 | | 0.063 | 0.012 |  |
| Myc | myelocytomatosis oncogene | | 0.130 | | 0.478 | 0.020 |  |
| Uaca | uveal autoantigen with coiled-coil domains and ankyrin repeats, transcript variant X1 | | 0.098 | | 0.370 | 0.04 |  |
| Dapk2 | death-associated protein kinase 2, transcript variant X1 | | 0.111 | | 0.073 | 0.02 |  |
| Rhbdd1 | rhomboid domain containing 1, transcript variant X1 | | 2.218 | | 0.055 | 0.055 |  |
| Col4a3 | collagen type IV alpha 3 chain, transcript variant X1 | | 0.051 | | 0.051 | 0.051 |  |
| Pim2 | Pim-2 proto-oncogene, serine/threonine kinase | | 0.448 | | 0.304 | 0.018 |  |
| Bex2 | brain expressed X-linked 2 | | 0.04 | | 0.129 | 0.04 |  |
| Defb1 | defensin beta 1 | | 1.707 | | 8.002 | 0.025 |  |
| Defa5 | defensin alpha 5, transcript variant X1 | | 0.042 | | 0.01 | 0.003 |  |
| Defa7 | defensin alpha 7 | | 0.040 | | 0.040 | 0.040 |  |
| Defa8 | defensin alpha 8 | | 1.000 | | 1.000 | 1.000 |  |
| Defa10 | defensin alpha 10 | | 0.384 | | 0.384 | 0.384 |  |
| Defa11 | defensin alpha 11, transcript variant X1 | | 0.05 | | 0.09 | 0.007 |  |
| RatNP-3b | defensin RatNP-3 precursor | | 0.012 | | 0.02 | 0.008 |  |
| **Cell death** | | | | | | |  |
| Pdcd6 | programmed cell death 6 | | 3.089 | | 2.876 | 0.123 |  |
| Rps6ka2 | ribosomal protein S6 kinase polypeptide 2, transcript variant X2 | | 0.071 | | 0.288 | 0.04 |  |
| Pdcd2 | programmed cell death 2 | | 7.108 | | 6.916 | 0.003 |  |
| Tgfb1 | transforming growth factor, beta 1 | | 2.553 | | 4.035 | 24.886 |  |
| Map3k10 | mitogen activated protein kinase kinase kinase 10 | | 0.031 | | 0.031 | 0.031 |  |
| Apbb1 | amyloid beta precursor protein binding family B member 1, transcript variant X2 | | 0.251 | | 0.036 | 0.04 |  |
| Prkcb | protein kinase C, beta, transcript variant X1 | | 0.155 | | 0.204 | 0.04 |  |
| Atp2a1 | ATPase sarcoplasmic/endoplasmic reticulum Ca2+ transporting 1 | | 0.076 | | 0.065 | 0.03 |  |
| Prdx5 | peroxiredoxin 5 | | 13.007 | | 13.406 | 3.311 |  |
| Sgms1 | sphingomyelin synthase 1 | | 2.495 | | 0.475 | 0.046 |  |
| Pdcd4 | programmed cell death 4, transcript variant X5 | | 0.014 | | 0.417 | 0.014 |  |
| Cyfip2 | cytoplasmic FMR1 interacting protein 2 | | 0.344 | | 0.458 | 0.03 |  |
| Mapk9 | mitogen-activated protein kinase 9, transcript variant 2 | | 3.259 | | 2.373 | 2.018 |  |
| Vdac1 | voltage-dependent anion channel 1 | | 0.492 | | 0.405 | 0.141 |  |
| Trpv2 | transient receptor potential cation channel, subfamily V, member 2, transcript variant 1 | | 97.846 | | 107.928 | 394.657 |  |
| Pmp22 | peripheral myelin protein 22 | | 0.131 | | 0.111 | 0.201 |  |
| Aldoc | aldolase, fructose-bisphosphate C | | 0.02 | | 0.036 | 0.02 |  |
| Pdk2 | pyruvate dehydrogenase kinase 2 | | 0.294 | | 0.271 | 0.03 |  |
| Ngfr | nerve growth factor receptor, transcript variant X1 | | 0.344 | | 0.334 | 0.046 |  |
| Mapt | microtubule-associated protein tau | | 0.142 | | 0.037 | 0.001 |  |
| Prkca | protein kinase C, alpha, transcript variant X1 | | 0.007 | | 0.037 | 0.07 |  |
| Axin2 | axin 2 | | 0.017 | | 0.017 | 0.017 |  |
| Nptx1 | neuronal pentraxin 1 | | 0.016 | | 0.016 | 0.016 |  |
| Aatk | apoptosis-associated tyrosine kinase, transcript variant X1 | | 0.144 | | 0.051 | 0.06 |  |
| Robo2 | roundabout guidance receptor 2, transcript variant X3 | | 2.423 | | 0.025 | 0.025 |  |
| Casr | calcium-sensing receptor, transcript variant X2 | | 0.043 | | 0.043 | 0.043 |  |
| Aifm3 | apoptosis inducing factor, mitochondria associated 3, transcript variant X2 | | 0.022 | | 0.022 | 0.022 |  |
| Brca2 | BRCA2, DNA repair associated, transcript variant X1 | | 0.013 | | 0.301 | 0.013 |  |
| Rnf34 | ring finger protein 34, transcript variant X2 | | 0.286 | | 0.445 | 0.01 |  |
| Taok3 | TAO kinase 3 | | 0.133 | | 0.242 | 0.05 |  |
| G0s2 | G0/G1switch 2 | | 96.375 | | 10.424 | 16.015 |  |
| Parm1 | prostate androgen-regulated mucin-like protein 1 | | 2.002 | | 4.422 | 0.021 |  |
| Cxcl2 | C-X-C motif chemokine ligand 2 | | 14525.877 | | 4248.301 | 2841.707 |  |
| Acin1 | apoptotic chromatin condensation inducer 1, transcript variant X5 | | 0.457 | | 0.411 | 0.05 |  |
| Gjb6 | gap junction protein, beta 6, transcript variant X5 | | 0.012 | | 0.331 | 0.012 |  |
| Clu | clusterin | | 9.011 | | 7.174 | 2.558 |  |
| Nkx3-1 | NK3 homeobox 1 | | 0.061 | | 0.061 | 0.061 |  |
| Tnfrsf10b | tumor necrosis factor receptor superfamily, member 10b | | 0.040 | | 0.040 | 0.040 |  |
| Slc25a4 | solute carrier family 25 member 4 | | 0.398 | | 0.405 | 0.318 |  |
| Ctsl | cathepsin L | | 6.653 | | 6.804 | 4.260 |  |
| Ryr2 | ryanodine receptor 2, transcript variant 2 | | 0.020 | | 0.111 | 0.020 |  |
| Fbxo18 | F-box protein, helicase, 18, transcript variant X4 | | 2.581 | | 2.214 | 3.550 |  |
| Sra1 | steroid receptor RNA activator 1 | | 3.460 | | 2.608 | 2.617 |  |
| Acaa2 | acetyl-CoA acyltransferase 2 | | 0.191 | | 0.494 | 0.04 |  |
| Mt3 | metallothionein 3 | | 0.131 | | 0.307 | 0.01 |  |
| Hmox1 | heme oxygenase 1 | | 9.578 | | 22.228 | 14.609 |  |
| Tox3 | TOX high mobility group box family member 3, transcript variant X1 | | 0.013 | | 0.042 | 0.013 |  |
| Siah1 | siah E3 ubiquitin protein ligase 1 | | 0.361 | | 0.451 | 0.06 |  |
| Dnase2 | deoxyribonuclease II, lysosomal | | 8.005 | | 10.609 | 0.040 |  |
| Nol3 | nucleolar protein 3, transcript variant X1 | | 0.117 | | 0.134 | 0.07 |  |
| Zmat3 | zinc finger, matrin type 3 | | 3.026 | | 0.485 | 0.026 |  |
| Foxo1 | forkhead box O1 | | 3.565 | | 0.060 | 0.060 |  |
| Siah2 | siah E3 ubiquitin protein ligase 2 | | 2.629 | | 0.477 | 0.050 |  |
| S100a8 | S100 calcium binding protein A8, transcript variant X1 | | 0.491 | | 0.370 | 0.042 |  |
| Mllt11 | myeloid/lymphoid or mixed-lineage leukemia; translocated to, 11 | | 0.095 | | 0.131 | 0.03 |  |
| Casp6 | caspase 6, transcript variant X2 | | 0.138 | | 0.362 | 0.011 |  |
| Ddit4 | DNA-damage-inducible transcript 4 | | 15.813 | | 13.501 | 4.340 |  |
| Madd | MAP-kinase activating death domain | | 0.028 | | 0.358 | 0.028 |  |
| Apip | APAF1 interacting protein, transcript variant X1 | | 0.020 | | 0.188 | 0.020 |  |
| Rmdn3 | regulator of microtubule dynamics 3 | | 0.441 | | 0.141 | 0.010 |  |
| Capn3 | calpain 3, transcript variant X1 | | 0.044 | | 0.095 | 0.07 |  |
| Il1b | interleukin 1 beta | | 4257.707 | | 1057.356 | 788.250 |  |
| Ptgis | prostaglandin I2 (prostacyclin) synthase | | 0.003 | | 0.181 | 0.03 |  |
| Snca | synuclein alpha | | 0.003 | | 0.032 | 0.003 |  |
| Mcm2 | minichromosome maintenance complex component 2 | | 0.342 | | 2.471 | 0.09 |  |
| Magi1 | membrane associated guanylate kinase, WW and PDZ domain containing 1, transcript variant X4 | | 0.061 | | 0.061 | 0.061 |  |
| Cidec | cell death-inducing DFFA-like effector c, transcript variant 3 | | 0.033 | | 0.033 | 0.033 |  |
| Gapdh | glyceraldehyde-3-phosphate dehydrogenase, transcript variant X1 | | 0.348 | | 0.495 | 0.003 |  |
| Epha7 | Eph receptor A7, transcript variant X1 | | 0.035 | | 0.035 | 0.035 |  |
| Xpa | XPA, DNA damage recognition and repair factor | | 0.362 | | 0.490 | 0.018 |  |
| Jun | Jun proto-oncogene, AP-1 transcription factor subunit | | 3.511 | | 2.881 | 8.080 |  |
| Faf1 | Fas associated factor 1 | | 0.156 | | 0.336 | 0.05 |  |
| Tnfrsf14 | TNF receptor superfamily member 1B | | 0.011 | | 0.046 | 0.011 |  |
| Birc6 | baculoviral IAP repeat-containing 6 | | 0.177 | | 0.382 | 0.08 |  |
| Rhob | ras homolog family member B | | 4.858 | | 2.470 | 0.03 |  |
| Egln3 | egl-9 family hypoxia-inducible factor 3 | | 3.043 | | 0.375 | 0.09 |  |
| Six1 | SIX homeobox 1 | | 0.104 | | 0.278 | 0.07 |  |
| Psen1 | presenilin 1, transcript variant X1 | | 0.022 | | 0.124 | 0.022 |  |
| Ddit3 | DNA-damage inducible transcript 3, transcript variant X2 | | 2.066 | | 3.676 | 3.846 |  |
| Ebag9 | estrogen receptor binding site associated, antigen, 9 | | 0.353 | | 0.455 | 0.05 |  |
| Tnfrsf11b | TNF receptor superfamily member 11B | | 0.060 | | 0.063 | 0.012 |  |
| Myc | myelocytomatosis oncogene | | 0.130 | | 0.478 | 0.020 |  |
| Nr4a1 | nuclear receptor subfamily 4, group A, member 1, transcript variant X2 | | 2.151 | | 2.378 | 0.05 |  |
| Uaca | uveal autoantigen with coiled-coil domains and ankyrin repeats, transcript variant X1 | | 0.098 | | 0.370 | 0.004 |  |
| Dapk2 | death-associated protein kinase 2, transcript variant X1 | | 0.111 | | 0.073 | 0.002 |  |
| Rhbdd1 | rhomboid domain containing 1, transcript variant X1 | | 2.218 | | 0.055 | 0.055 |  |
| Col4a3 | collagen type IV alpha 3 chain, transcript variant X1 | | 0.051 | | 0.051 | 0.051 |  |
| Pim2 | Pim-2 proto-oncogene, serine/threonine kinase | | 0.448 | | 0.304 | 0.018 |  |
| Bex2 | brain expressed X-linked 2 | | 0.004 | | 0.129 | 0.004 |  |
| S100a13 | S100 calcium binding protein A13 | | 0.457 | | 0.485 | 0.003 |  |
| S100a16 | S100 calcium binding protein A16 | | 1.535 | | 1.566 | 0.002 |  |
| S100a3 | S100 calcium binding protein A3, transcript variant X2 | | 0.021 | | 0.513 | 0.021 |  |
| S100a6 | S100 calcium binding protein A6, transcript variant X1 | | 1.928 | | 2.637 | 0.943 |  |
| S100a8 | S100 calcium binding protein A8, transcript variant X1 | | 0.42 | | 0.370 | 0.042 |  |
| S100a9 | S100 calcium binding protein A9, transcript variant X1 | | 0.510 | | 0.267 | 0.041 |  |
| Tnf | tumor necrosis factor | | 117.150 | | 60.404 | 0.954 |  |
| Smad4 | SMAD family member 4 | | 1.471 | | 1.439 | 0.02 |  |
| Defb1 | defensin beta 1 | | 1.707 | | 8.002 | 0.025 |  |
| Defa5 | defensin alpha 5, transcript variant X1 | | 0.042 | | 0.01 | 0.003 |  |
| Defa7 | defensin alpha 7 | | 0.040 | | 0.040 | 0.040 |  |
| Defa8 | defensin alpha 8 | | 1.000 | | 1.000 | 1.000 |  |
| Defa10 | defensin alpha 10 | | 0.384 | | 0.384 | 0.384 |  |
| Defa11 | defensin alpha 11, transcript variant X1 | | 0.05 | | 0.09 | 0.007 |  |
| RatNP-3b | defensin RatNP-3 precursor | | 0.012 | | 0.02 | 0.008 |  |
| **Immune response** | | | | | | |  |
| Tgfb1 | | transforming growth factor, beta 1 | 2.553 | 4.035 | | 24.886 |  |
| Ffar2 | | free fatty acid receptor 2, transcript variant X5 | 0.279 | 0.023 | | 0.023 |  |
| Gp2 | | glycoprotein 2, transcript variant X1 | 0.018 | 0.221 | | 0.003 |  |
| Prkcb | | protein kinase C, beta, transcript variant X1 | 0.155 | 0.204 | | 0.004 |  |
| Mylpf | | myosin light chain, phosphorylatable, fast skeletal muscle | 0.128 | 0.098 | | 0.045 |  |
| Ctf1 | | cardiotrophin 1, transcript variant X1 | 0.011 | 0.045 | | 0.011 |  |
| Irf7 | | interferon regulatory factor 7, transcript variant X2 | 14.265 | 3.406 | | 12.200 |  |
| Ms4a2 | | membrane spanning 4-domains A2 | 0.015 | 0.015 | | 0.015 |  |
| Nfkb2 | | nuclear factor kappa B subunit 2, transcript variant X1 | 3.489 | 3.749 | | 3.428 |  |
| Stx8 | | syntaxin 8, transcript variant X1 | 0.018 | 0.434 | | 0.010 |  |
| Tnk1 | | tyrosine kinase, non-receptor, 1, transcript variant X6 | 0.033 | 0.394 | | 0.033 |  |
| Lgals9 | | galectin 9 | 0.281 | 0.301 | | 0.002 |  |
| Ngfr | | nerve growth factor receptor, transcript variant X1 | 0.344 | 0.334 | | 0.046 |  |
| Milr1 | | mast cell immunoglobulin-like receptor 1, transcript variant X1 | 0.113 | 0.058 | | 0.033 |  |
| Tnk2 | | tyrosine kinase, non-receptor, 2, transcript variant X10 | 0.315 | 0.283 | | 0.002 |  |
| Rnf168 | | ring finger protein 168, E3 ubiquitin protein ligase | 0.299 | 0.145 | | 0.007 |  |
| Dlg1 | | discs large MAGUK scaffold protein 1 | 0.050 | 0.246 | | 0.050 |  |
| Vpreb2 | | pre-B lymphocyte gene 2 | 0.012 | 0.012 | | 0.012 |  |
| Igll1 | | immunoglobulin lambda-like polypeptide 1, transcript variant X1 | 0.021 | 0.021 | | 0.021 |  |
| Fcer1a | | Fc fragment of IgE receptor Ia, transcript variant X2 | 0.061 | 0.061 | | 0.061 |  |
| Cxcl10 | | C-X-C motif chemokine ligand 10 | 24.800 | 5.373 | | 17.210 |  |
| Cxcl2 | | C-X-C motif chemokine ligand 2 | 121 | 158 | | 126 |  |
| Cxcl1 | | chemokine (C-X-C motif) ligand 1 | 217.400 | 97.775 | | 58.512 |  |
| Pf4 | | platelet factor 4 | 3.854 | 5.853 | | 9.431 |  |
| Tlr6 | | toll-like receptor 6 | 6.311 | 2.044 | | 0.031 |  |
| Peli1 | | pellino E3 ubiquitin protein ligase 1 | 2.235 | 0.365 | | 0.012 |  |
| Peli2 | | pellino E3 ubiquitin protein ligase family member 2 | 0.233 | 0.266 | | 0.026 |  |
| Rnase2 | | ribonuclease A family member 2 | 0.061 | 0.018 | | 0.018 |  |
| Ctsg | | cathepsin G, transcript variant X1 | 0.04 | 0.05 | | 0.01 |  |
| Tnfrsf10b | | tumor necrosis factor receptor superfamily, member 10b | 0.040 | 0.040 | | 0.040 |  |
| Sftpd | | surfactant protein D | 9.154 | 4.692 | | 4.866 |  |
| Star | | Janus kinase 3, transcript variant X2 | 0.033 | 0.033 | | 0.033 |  |
| LOC684871 | | steroidogenic acute regulatory protein | 3.731 | 3.236 | | 7.611 |  |
| Np4 | | defensin NP-4 precursor, transcript variant X1 | 0.062 | 0.08 | | 0.03 |  |
| Ctsl | | RAB20, member RAS oncogene family | 6.653 | 6.804 | | 4.260 |  |
| Cxcl14 | | C-X-C motif chemokine ligand 14 | 0.450 | 0.219 | | 0.001 |  |
| Edn1 | | endothelin 1, transcript variant X1 | 0.023 | 0.023 | | 0.023 |  |
| Wrnip1 | | Werner helicase interacting protein 1 | 0.436 | 0.192 | | 0.006 |  |
| Irf4 | | interferon regulatory factor 4 | 0.022 | 0.311 | | 0.022 |  |
| Kif5b | | kinesin family member 5B, transcript variant X1 | 0.145 | 0.439 | | 0.003 |  |
| Lyst | | lysosomal trafficking regulator | 0.013 | 0.216 | | 0.013 |  |
| Ankhd1 | | ankyrin repeat and KH domain containing 1 | 0.005 | 0.132 | | 0.003 |  |
| Cd14 | | CD14 molecule, transcript variant X1 | 11.958 | 6.473 | | 12.230 |  |
| Il15 | | interleukin 15, transcript variant X6 | 0.038 | 0.038 | | 0.038 |  |
| Plcg2 | | phospholipase C, gamma 2, transcript variant X1 | 0.011 | 0.362 | | 0.011 |  |
| Cyba | | cytochrome b-245 alpha chain | 4.993 | 7.006 | | 2.001 |  |
| Gapt | | Grb2-binding adaptor protein, transmembrane, transcript variant X2 | 5.204 | 0.374 | | 0.030 |  |
| Ptx3 | | pentraxin 3 | 15.644 | 17.861 | | 202.385 |  |
| Sema4a | | semaphorin 4A, transcript variant X3 | 4.859 | 4.000 | | 4.184 |  |
| S100a8 | | S100 calcium binding protein A8, transcript variant X1 | 0.491 | 0.370 | | 0.042 |  |
| Polr3c | | polymerase (RNA) III subunit C, transcript variant X2 | 0.030 | 2.277 | | 0.017 |  |
| Chia | | chitinase, acidic | 9.446 | 5.649 | | 13.709 |  |
| Stxbp3 | | syntaxin binding protein 3 | 2.657 | 0.422 | | 0.016 |  |
| Trim10 | | tripartite motif-containing 10 | 0.024 | 0.024 | | 0.024 |  |
| RT1-CE5 | | RT1 class I, locus CE5, transcript variant X1 | 3.231 | 3.304 | | 2.097 |  |
| Vpreb3 | | pre-B lymphocyte 3 | 0.014 | 0.014 | | 0.014 |  |
| Il1r n | | interleukin 1 receptor antagonist, transcript variant X1 | 38.335 | 21.056 | | 3.521 |  |
| Prg2 | | proteoglycan 2 | 0.013 | 0.001 | | 0.006 |  |
| Prg3 | | proteoglycan 3, pro eosinophil major basic protein 2, transcript variant X1 | 0.002 | 0.002 | | 0.002 |  |
| Rag1 | | recombination activating gene 1 | 0.014 | 0.322 | | 0.014 |  |
| Il1b | | interleukin 1 beta | 42.707 | 64.356 | | 78.250 |  |
| Bpifb1 | | BPI fold containing family B, member 1, transcript variant X2 | 0.022 | 0.001 | | 0.001 |  |
| Ahcy | | adenosylhomocysteinase | 3.882 | 2.946 | | 2.459 |  |
| Slpi | | secretory leukocyte peptidase inhibitor | 151.242 | 124.681 | | 25.326 |  |
| Cd36 | | CD36 molecule | 0.035 | 0.035 | | 0.035 |  |
| RGD1565355 | | similar to fatty acid translocase/CD36 | 0.038 | 0.267 | | 0.021 |  |
| Zyx | | zyxin, transcript variant X4 | 3.773 | 2.799 | | 2.738 |  |
| Snca | | synuclein alpha | 0.003 | 0.032 | | 0.003 |  |
| A2m | | alpha-2-macroglobulin | 0.324 | 0.240 | | 0.068 |  |
| C1r | | complement C1r subcomponent | 3.617 | 3.622 | | 2.908 |  |
| C1s | | complement component 1, s subcomponent | 11.807 | 6.021 | | 3.837 |  |
| Gapdh | | glyceraldehyde-3-phosphate dehydrogenase, transcript variant X1 | 0.348 | 0.495 | | 0.003 |  |
| Itfg2 | | integrin alpha FG-GAP repeat containing 2 | 0.055 | 0.269 | | 0.055 |  |
| Ccl21 | | C-C motif chemokine ligand 21 | 22.585 | 18.812 | | 33.690 |  |
| Sit1 | | signaling threshold regulating transmembrane adaptor 1 | 0.211 | 0.018 | | 0.018 |  |
| Mtor | | mechanistic target of rapamycin | 0.434 | 0.333 | | 0.007 |  |
| Tnfrsf14 | | TNF receptor superfamily member 14, transcript variant X6 | 0.011 | 0.046 | | 0.011 |  |
| Eif2ak2 | | eukaryotic translation initiation factor 2-alpha kinase 2, transcript variant X2 | 4.425 | 2.301 | | 14.835 |  |
| Adam17 | | ADAM metallopeptidase domain 17 | 2.304 | 2.349 | | 6.639 |  |
| Psen1 | | presenilin 1, transcript variant X1 | 0.022 | 0.124 | | 0.022 |  |
| Chga | | chromogranin A | 0.007 | 0.007 | | 0.007 |  |
| Serpina3n | | serine (or cysteine) peptidase inhibitor, clade A, member 3N | 2.472 | 65.703 | | 45.430 |  |
| Evl | | Enah/Vasp-like | 0.482 | 0.430 | | 0.004 |  |
| Elane | | elastase, neutrophil expressed | 0.111 | 0.004 | | 0.004 |  |
| Tnfrsf11b | | TNF receptor superfamily member 11B | 0.060 | 0.063 | | 0.012 |  |
| Enpp2 | | ectonucleotide pyrophosphatase/phosphodiesterase 2 | 0.202 | 0.149 | | 0.003 |  |
| Myc | | myelocytomatosis oncogene | 0.130 | 0.478 | | 0.020 |  |
| Sla | | src-like adaptor | 0.024 | 0.300 | | 0.024 |  |
| Pla2g6 | | phospholipase A2 group VI, transcript variant X8 | 0.369 | 0.213 | | 0.011 |  |
| Cadm1 | | cell adhesion molecule 1 | 0.308 | 0.486 | | 0.189 |  |
| Il18 | | interleukin 18 | 4.362 | 3.055 | | 2.196 |  |
| Syncrip | | synaptotagmin binding, cytoplasmic RNA interacting protein, transcript variant X4 | 0.048 | 2.872 | | 0.027 |  |
| C3 | | complement component 3 | 6.790 | 3.565 | | 2.383 |  |
| Slc11a1 | | solute carrier family 11 member 1, transcript variant X1 | 47.177 | 66.479 | | 101.538 |  |
| Tlr7 | | toll-like receptor 7, transcript variant X2 | 0.077 | 0.076 | | 0.043 |  |
| Btk | | Bruton tyrosine kinase | 0.020 | 0.249 | | 0.020 |  |
| Defb1 | | defensin beta 1 | 1.707 | 8.002 | | 0.025 |  |
| Defa5 | | defensin alpha 5, transcript variant X1 | 0.042 | 0.01 | | 0.003 |  |
| Defa7 | | defensin alpha 7 | 0.040 | 0.040 | | 0.040 |  |
| Defa8 | | defensin alpha 8 | 1.000 | 1.000 | | 1.000 |  |
| Defa10 | | defensin alpha 10 | 0.384 | 0.384 | | 0.384 |  |
| Defa11 | | defensin alpha 11, transcript variant X1 | 0.05 | 0.09 | | 0.007 |  |
| RatNP-3b | | defensin RatNP-3 precursor | 0.012 | 0.02 | | 0.008 |  |
| Inflammatory response | | | | | | | |
| Fpr1 | | formyl peptide receptor 1, transcript variant X1 | 0.018 | 0.018 | | 0.018 | |
| Tgfb1 | | transforming growth factor, beta 1 | 2.553 | 4.035 | | 24.886 | |
| Ffar2 | | free fatty acid receptor 2, transcript variant X5 | 0.279 | 0.023 | | 0.023 | |
| Cebpa | | CCAAT/enhancer binding protein alpha, transcript variant 1 | 0.244 | 2.285 | | 0.104 | |
| Ms4a2 | | membrane spanning 4-domains A2 | 0.015 | 0.015 | | 0.015 | |
| Sgms1 | | sphingomyelin synthase 1 | 2.495 | 0.475 | | 0.046 | |
| Nfkb2 | | nuclear factor kappa B subunit 2, transcript variant X1 | 3.489 | 3.749 | | 3.428 | |
| Adra2a | | adrenoceptor alpha 2A | 3.707 | 8.098 | | 7.106 | |
| Clec10a | | C-type lectin domain family 10, member A | 2.218 | 5.715 | | 2.627 | |
| Lgals9 | | galectin 9 | 0.281 | 0.301 | | 0.002 | |
| Ngfr | | nerve growth factor receptor, transcript variant X1 | 0.344 | 0.334 | | 0.046 | |
| Tfr2 | | transferrin receptor 2 | 0.047 | 0.047 | | 0.047 | |
| Cxcl2 | | C-X-C motif chemokine ligand 2 | 4120.21 | 4248.301 | | 2841.707 | |
| Cxcl1 | | chemokine (C-X-C motif) ligand 1 | 217.400 | 97.775 | | 58.512 | |
| Pf4 | | platelet factor 4 | 3.854 | 5.853 | | 9.431 | |
| Tnfrsf10b | | tumor necrosis factor receptor superfamily, member 10b | 0.040 | 0.040 | | 0.040 | |
| Prkcq | | protein kinase C, theta | 0.009 | 0.190 | | 0.009 | |
| Cd14 | | CD14 molecule, transcript variant X1 | 11.958 | 6.473 | | 12.230 | |
| Hmox1 | | heme oxygenase 1 | 9.578 | 22.228 | | 14.609 | |
| Il15 | | interleukin 15, transcript variant X6 | 0.038 | 0.038 | | 0.038 | |
| Cyba | | cytochrome b-245 alpha chain | 4.993 | 7.006 | | 2.001 | |
| S100a8 | | S100 calcium binding protein A8, transcript variant X1 | 0.491 | 0.370 | | 0.042 | |
| Defb1 | | defensin beta 1 | 1.707 | 8.002 | | 0.025 | |
| Defa5 | | defensin alpha 5, transcript variant X1 | 0.042 | 0.01 | | 0.003 | |
| Defa7 | | defensin alpha 7 | 0.080 | 0.090 | | 0.040 | |
| Defa10 | | defensin alpha 10 | 0.484 | 0.384 | | 0.310 | |
| Defa11 | | defensin alpha 11, transcript variant X1 | 0.050 | 0.090 | | 0.070 | |
| RatNP-3b | | defensin RatNP-3 precursor | 0.012 | 0.200 | | 0.080 | |
| Alox5 | | arachidonate 5-lipoxygenase, transcript variant X1 | 0.015 | 0.186 | | 0.015 | |
| A2m | | alpha-2-macroglobulin | 0.324 | 0.240 | | 0.068 | |
| Ccl21 | | C-C motif chemokine ligand 21 | 22.585 | 18.812 | | 33.690 | |
| Plaa | | phospholipase A2, activating protein | 0.499 | 0.430 | | 0.008 | |
| Park7 | | Parkinsonism associated deglycase, transcript variant 1 | 0.245 | 0.426 | | 0.002 | |
| Tnfrsf14 | | TNF receptor superfamily member 14, transcript variant X6 | 0.011 | 0.046 | | 0.011 | |
| Sdc1 | | syndecan 1 | 2.776 | 5.676 | | 8.443 | |
| Adam17 | | ADAM metallopeptidase domain 17 | 2.304 | 2.349 | | 6.639 | |
| Hif1a | | hypoxia inducible factor 1 alpha subunit, transcript variant X1 | 7.320 | 4.631 | | 10.997 | |
| Serpina1 | | serpin family A member 1 | 0.061 | 0.061 | | 0.061 | |
| Serpina3n | | serine (or cysteine) peptidase inhibitor, clade A, member 3N | 2.472 | 65.703 | | 45.430 | |
| Elane | | elastase, neutrophil expressed | 0.111 | 0.004 | | 0.004 | |
| Tnfrsf11b | | TNF receptor superfamily member 11B | 0.060 | 0.063 | | 0.012 | |
| C3 | | complement component 3 | 6.790 | 3.565 | | 2.383 | |
| Slc11a1 | | solute carrier family 11 member 1, transcript variant X1 | 47.177 | 66.479 | | 101.538 | |
| Ugt1a1 | | UDP glucuronosyltransferase family 1 member A1 | 2.102 | 5.903 | | 0.007 | |
| Btk | | Bruton tyrosine kinase | 0.020 | 0.249 | | 0.020 | |
